# Closing the Nutrient Loop—The New Approaches to Recovering Biomass Minerals during the Biorefinery Processes

**DOI:** 10.3390/ijerph20032096

**Published:** 2023-01-23

**Authors:** Diana Constantinescu-Aruxandei, Florin Oancea

**Affiliations:** Department of Bioresources, Bioproducts Group, National Institute for Research & Development in Chemistry and Petrochemistry—ICECHIM, Splaiul Independenței nr. 202, Sector 6, 060021 Bucharest, Romania

**Keywords:** biomass, mineral, recovery, pre-treatment, fertilizer

## Abstract

The recovery of plant mineral nutrients from the bio-based value chains is essential for a sustainable, circular bioeconomy, wherein resources are (re)used sustainably. The widest used approach is to recover plant nutrients on the last stage of biomass utilization processes—e.g., from ash, wastewater, or anaerobic digestate. The best approach is to recover mineral nutrients from the initial stages of biomass biorefinery, especially during biomass pre-treatments. Our paper aims to evaluate the nutrient recovery solutions from a trans-sectorial perspective, including biomass processing and the agricultural use of recovered nutrients. Several solutions integrated with the biomass pre-treatment stage, such as leaching/bioleaching, recovery from pre-treatment neoteric solvents, ionic liquids (ILs), and deep eutectic solvents (DESs) or integrated with hydrothermal treatments are discussed. Reducing mineral contents on silicon, phosphorus, and nitrogen biomass before the core biorefinery processes improves processability and yield and reduces corrosion and fouling effects. The recovered minerals are used as bio-based fertilizers or as silica-based plant biostimulants, with economic and environmental benefits.

## 1. Introduction

Biomass biorefinery converts biological materials into bio-based chemicals and biofuels [1,2]. Many technologies have been developed for biomass biorefinery, depending on the biomass used and the purpose employed: anaerobic digestion [3,4,5]; enzymatic processing and fermentation for biofuel and bio-based chemicals production [6,7]; fast and slow pyrolysis for bio-oil (pyrolysis oil) and biochar [8,9]; thermochemical conversion to syngas, followed by the Fischer–Tropsch process [10].

Lignocellulose feedstocks have a highly recalcitrant complex structure, wherein hydrophilic biopolymers, cellulose, and hemicellulose are closely bound to hydrophobic lignin [11,12,13]. The disruption of such a complex structure needs various types of pre-treatment [14,15]. Ideally, biomass’s organic and inorganic fractions should be separated entirely before or during biorefinery processing. Recovering and reusing minerals from biomass waste is essential for a sustainable, circular bioeconomy, wherein resources are (re)used [16,17,18]. Waste-based biomass generally has higher nitrogen (N), phosphorus (P), and potassium (K) contents than lignocellulosic plants; therefore, it is the more preferred feedstock for nutrient recovery [17]. Marine biomass and animal by-products used as feedstock in biorefinery contain high levels of minerals [19,20] that hamper the conversion into value-added products.

The widest-used approach is to recover plant nutrients in the last stage of biomass utilization processes—e.g., from ash, wastewater, or anaerobic digestate [21]. The innovative approach is to recover mineral nutrients, also from the initial stages of biomass biorefinery, especially during the biomass pre-treatment step. However, such an approach is hampered by the relatively low concentrations of nutrients, which negatively influences nutrient recovery [22].

The highest mineral content in biorefinery feedstocks is on the biomass of silica-accumulating plant species [23]. The biomass from the grasses of the *Poaceae* and *Cyperaceae* families is particularly interesting for biorefinery [11]. In rice straws, the silica content is higher than 10%, usually between 13–16% [24,25]. In rice husks, the silica content is even higher, reaching almost 20% [26]. Wheat straw accumulated up to 7.4% silica [27,28]. On other grass biomass feedstocks, e.g., napiergrass, *Cenchrus purpureus* (syn. *Pennisetum purpureum*), pearl millet-napiergrass hybrid, *Pennisetum glaucum* × *Pennisetum purpureum*, switchgrass, *Panicum virgatum*, energycane, *Saccharum* L. spp, miscanthus, *Miscanthus* × *giganteus*, perennial sorghum, *Sorghum* spp., pearl millet, *Pennisetum glaucum*, annual Sorghum, and *Sorghum bicolor* cultivated for two years in Texas, USA, accumulated from 2.68% (in napiergrass) to 6.74%, in annual sorghum [29]. The grass reeds, *Phragmites karka* and *Arundo donax*, with high potential as biorefinery feedstocks [30,31], accumulate from 1.64% silica (in *A. donax* culm) to 8% silica (in *A. donax* leaves) [32].

The average total nitrogen concentration in wheat straws ranges from 0.39% to 0.58%, depending on cultivar and fertilization conditions [33]. Rice straws from North China contain 1.22% total potassium, 0.65% total nitrogen, and 0.18% total phosphorus [34]. Silica-accumulating plants contain up to 2.37% potassium [35,36] and 0.1% phosphorus [37] in the silicified structures (phytoliths). Phytoliths from silicon-rich biomass were proposed to be used as slow-release potassium fertilizers [38].

The minerals in biomass can be divided into two categories: leachable and non-leachable. This delimitation is particularly important for some biomass types, such as sugarcane stalks or algae, for which the liquid fractions, such as juice or sap, are valorized [39,40], but also for cases in which water extraction could be applied. The leachable inorganics are extracted in these fractions.

Several hydrothermal pre-treatment factors were found to influence the levels and distributions of minerals in wheat straw biomass differently during the biorefining process [41]. Based on the results, the authors divided the constituents into two groups: group one (phosphorus, magnesium, potassium, manganese, zinc, and calcium), which was correlated (was associated) with hemicelluloses (xylose and arabinose) and was highly influenced by pH; group two (silicon, iron, copper, and aluminum), correlated with lignin and cellulose.

Other bioeconomy side-streams used as biorefinery feedstock with a high content of minerals are the by-products from chain values based on aquatic organisms. Fish scales and fish bones, which are used to produce collagen hydrolysate with interesting biological properties [42,43,44,45], contain 4.24 ± 0.89% phosphorus and 5.36 ± 1.48% calcium [46] and, respectively, 9.2–9.8% phosphorus and 4.1–4.9% calcium [47].

For feedstocks with high mineral content, demineralization (and the further recovery of the nutrients to produce bio-based fertilizers) simplifies the biorefinery process. One possibility to recover mineral nutrients in the initial biorefinery stage is to use the technology developed to recover nutrients from waste streams [16]. The technologies for plant nutrients recovery from waste streams were classified 7 years ago into three categories, which could be used separately or combined: (1) nutrient accumulation/nutrient concentration (for waste streams with low nutrient concentration); (2) nutrient release (mainly for insoluble nutrient forms); (3) nutrient extraction. Nutrient accumulation can be performed with biological (bacterial, algae, or plant accumulation), chemical (coagulation, flocculation), and physical (adsorption/ion exchange, membrane filtration, magnetic separation) techniques [48]. Nutrient release technologies include biological release (anaerobic digestion, bioleaching/extraction), thermochemical stabilization, and chemical release (thermal hydrolysis, wet oxidation, incineration, gasification, and pyrolysis, acid, or base-induced leachate and several commercial processes that couple thermochemical stabilization with chemical extraction). Nutrient recovery and extraction can be accomplished by physicochemical methods (chemical precipitation/crystallization, such as the formation of struvite, use of gas-permeable membrane and absorption, electrodialysis (ED), liquid–gas stripping) [48]. Such technology could be adapted to the wet stage process of the biorefinery process, wherein fluid streams are generated [16].

Several existing technological approaches, such as the recovery of biosolids from anaerobic digestion, production of biochar and/or ash, aerobic composting, and phosphorus, and nitrogen recovery, were already reviewed to demonstrate the need for an integrated approach [16]. Nutrient recovery technology already reaches a technological readiness level higher (TRL) than 6—phosphorous precipitation, ash leaching, animal bone biochar, anaerobic digestion, and vermicomposting [21]. None of these are easy to translate to the initial stages of biorefinery, especially to the new developments, such as the use of neoteric solvents (ionic liquid and deep eutectic solvents) or the new enzymes, such as lytic polysaccharide monooxygenases (LPMO), which has potential to recover mineral nutrients. Until now, such mature technologies were used to convert biorefinery by-products and not to recover mineral nutrients in the initial biorefinery stages.

The nutrient recovery was focused mainly on nitrogen and phosphorus. Synthetic nitrogen fertilizers are mineral nutrients with high production costs and environmental impact [49,50]. Phosphorus is a nutrient with a shortage risk in a “telecoupled planet” [51].

Silicon recovery from biomass during the biorefinery process, especially during the initial stage, was not yet reviewed. Silicon is highly accumulated in monocots and pteridophytes [52]. By-products from monocot plants cultivation (wheat and barley straw, corn stalk and corn cobs, sugar cane and sweets sorghum bagasse, excess aquatic reed biomass, etc.) are considered important raw materials for biorefinery [53,54]. Depending on its local availability, the type of lignocellulosic biomass varies between regions. In Europe, wheat straw is the most abundant source of biomass (almost 45% of the total cereal crop production was wheat in 2014) [55]. Silicon, usually present in plant lignocellulosic material in various forms of biosilica, complicates the biorefinery process [56,57]. The uncontrolled precipitation of silica scale in industrial installations is a significant risk associated with processing silica-rich biomass [58]. Hydrolytic enzymes activities are hampered by biosilica [57,59]. The recovery of silicon from biomass during the initial stages is benefic for further processing. Biosilica return to soil is essential for soil health [60]. Silicon was recently proposed to be included among plant macronutrients [61]. Silicon plant protectant and plant biostimulant activity are broadly accepted [60,62,63,64]. Soil bioavailable silicon pool is mainly based on biosilica [65,66].

The recovery of the silicon, nitrogen, and phosphorus anionic nutrient species also leads to the recovery of cationic nutrients—Ca^2+^, Mg^2+^, and microelements. The recovery of sulfur is significant for anaerobic digestion optimization.

**Figure 1:**
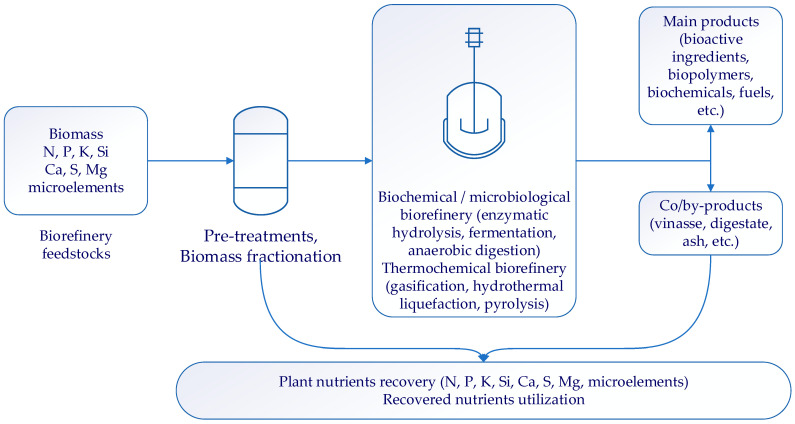
Ilustration of the dual goal followed in this work: nutrient recovery solutions during the biorefinery processes and recovered nutrients utilization.

Our paper aims to evaluate the nutrient recovery solutions from a trans-sectorial perspective, including biomass processing and the agricultural use of recovered nutrients. Each technology related to mineral nutrients recovery from biomass has its own advantages and challenges and has reached different technology readiness levels, from laboratory level (ED, gas-permeable membranes) to mature commercial technologies [48]. The potential of the already developed biomass processing technologies to incorporate solutions related to nutrients recovery are analyzed after the description of such technologies. The most promising approach is most likely to be integrated systems consisting of several complementary and inter-dependent techniques that reduce energy and water (re)use and recover (bio)fertilizers with lower chemical and physical hazards—Figure 1.

## 2. Recent Approaches for Mineral Recovery That Could Be Used during the First Stages of Biomass Biorefinery

The minerals from the side-stream biomass could have various applications, either in agriculture or in industry. These minerals could be recovered either at the beginning or at the end of biomass processing. The most advanced and mature current technologies are related to the second approach. For an optimal biorefinery process, more and more studies show that the minerals also need to be separated from the first stages, as minerals negatively interfere with the following processes, such as biofuel production, solvent recovery, biomolecules properties, and/or induce corrosion and biofouling, etc. In some cases, depending also on the biomass type, a mixture of initial and final stages might be necessary for the full and optimal recovery of minerals. In this chapter, we briefly review some of the recently proposed solutions for mineral recovery that are used, or could be used, at the first stages in biorefinery. Some of the methods that are used as pre-treatment for organic biomass destructuration, and fraction separation have the potential to also recover the minerals, but most of them have not been fully exploited. We present their current state-of-the-art status and discuss possible development and alternative approaches.

In this section, we briefly overview the current state-of-the-art of the methods that are, or could be, used also for mineral recovery, including the methods that currently mainly target the organic biomass fractionations. We discuss the possible utilization of these methods, either as a stand-alone or in combination with other methods, and integration into the biorefinery cascade approach. More specific information for Si, P, N, and S is given in the following chapters. We hope that discussing the processing of the mineral and organic fractions together will help the researchers to bring new ideas for the optimization of biorefinery technologies from the techno-economical and environmental point-of-view.

### 2.1. Dry Separation Processes

In the last decade, processes that use physical separation of dry biomass were developed. Representative examples are turbo- and electrostatic separation. Turbo-separation separates particles according to their mass, density, and size. Electrostatic separation is separation based on the electrostatic surface properties—electrical charges related to the chemical composition of the surface. Combined turbo- and electrostatic separation were used to separate rice straw’s organic and mineral fractions. In specific conditions, the combined processes partially separate the organic component from the minerals [67].

These turbo- and electrostatic dry separation techniques have the potential to separate mineral fractions from the organic ones, since the first stage of the biorefinery. The potential of such physical dry separation methods was demonstrated in the separations of proteins [68,69,70], grape stalk fibres disentangling [71], or arabinoxylans concentration from wheat bran [72]. The biomass pre-treatment [73] and the design of the equipment [74] significantly influence the process. The separation of the biomass fractions of interest, such as proteins, determines, in the remaining fraction, a concentration of the minerals in remaining fractions. For example, in the case of tribo-electrostatic the separation of the protein from sunflower or rapeseed meal, the fraction enriched in the proteins has around half of the initial mineral content [75]. Further developments in this direction are expected in the following years.

### 2.2. Wet Treatments

The wet treatments are more investigated than the dry ones, probably because they represent a continuation of the classical approaches, such as the wet acid-based pre-treatment for biomass destructuration. The acid-based pre-treatment is still the focus of many researchers, and its advantages and disadvantages were recently reviewed, together with the importance of mineral removal before biofuel production [76]. Sulphuric acid, for example, is efficient in extracting inorganic material, mainly alkaline (Na, K) minerals. An alternative to a wet acid-based method could be the relatively recently developed techniques known as dry dilute acid solution pre-treatment (DDAP), which exclude the presence of free-moving water using a solid:liquid ratio of 2:1, recently reviewed by Hoang et al. [76]. The method should present an advantage, compared with the classical wet technology, with respect to the impact on the environment. This method has just started to be explored for biomass pre-treatment, and the efficiency on mineral recovery has yet to be investigated. The only information available, in this respect, was provided by Zhou et al., who reported significant amounts of nutrient salts of corn stover hydrolisate, mostly sufficient for citric acid production by *Aspergillus niger* fermentation, but after dry dilution acid pre-treatment and hydrolisation by cellulase [77]. The addition of certain minerals was detrimental to the fermentation. It is not clear how many minerals the pre-treatment removed or if removal of extra minerals would have benefitted the fermentation.

In the case of some marine types of biomass, such as algae, direct and easy mineral recovery can be obtained by the direct squeeze of sap out of the fresh biomass or from the supernatant after applying an additional step of valuable protein recovery by ammonium sulphate precipitation, as reported by Baghel et al. [40,78]. The liquid fractions were found to be rich in minerals.

Other wet methods are based on microbial and enzymatic processes, but they were relatively recently and mainly applied to a silica-rich biomass chemical pre-treatments with alternative solvents, such as ionic liquids and deep eutectic solvents, which are also just starting to be explored from the mineral recovery point of view. Some of these methods were not initially aimed at mineral recovery, but at organic biomass processing. As the interest in the mineral issue has increased in the past years, it was found that, in some cases, the methods could be useful for this target. For this, it is important to understand the mechanism during the organic biomass fractionation and what happens with the mineral fractions, in particular, the organically-bound one through covalent or non-covalent bonds that constitutes 1–5% of the plant cell wall. For example, boron [79,80], calcium [81], silicon [56], and other references therein have been already shown to reside also in interactions with the organic biomass.

#### 2.2.1. Wet Pre-Treatments Based on Biological and Enzymatic Processes

Biological and biochemical pre-treatments of various sources of biomass have been the subject of significant attention over the past few years as more sustainable alternatives to thermochemical methods. The advantages of biological and biochemical methods, compared to thermal and chemical ones, are the lower toxicity and more ecological and cost-effective processes. The main attention has been given to the organic material destructuration, especially for the lignocellulose matrix and the effects on biogas, bio-oil, and bioethanol production. Several recent reviews on this topic are available [82,83,84,85]; therefore, it is not necessary to go more in depth in this respect. The pre-treatments involving either microbial growth and action or the use of enzymatic cocktails of hydrolytic and oxidative enzymes are the most advanced and in depth analyzed ones.

An important evolution occurred recently with the discovery [86] of what is thought to be the enzymes that act first on polysaccharides, the lytic polysaccharide monooxygenases (LPMO), members (AA9–11 and AA13) of the auxiliary activities (AA) family, and the copper enzymes that boost cellulose hydrolysis by oxidative cleavage of the glycosidic bond [87] before the attack of cellulases (reviewed in [88]). LPMOs seem to be active on numerous polysaccharides, such as chitin [86], cellulose [89], starch [90,91], xyloglucan, glucomannan, and cellodextrins [92,93,94]. For efficient activity, the enzymes need molecular oxygen and external electron donors [95], which can be redox enzymes [96,97], small molecules reductants [86,89], and/or photosynthetic pigments [98], depending on the microorganism lifestyle [99]. The current commercial enzyme cocktails, such as Cellic CTec2/3, contain also LPMOs, and the (pre)processing parameters have to be reconsidered, in order to optimize the overall enzymatic activity and cost-effective lignocellulose valorization [95,100], mainly because lignin was also shown to boost the LPMO activity [100,101].

Interestingly, the LPMOs AA11 genes were also found in the genome of the dermatophytic fungi [102]. It was proposed that some of these enzymes could be involved in keratin degradation [102,103], either by breaking the glycosylation bonds in the non-coiled head-structure of keratin filaments or by a reaction on tyrosine [103], but no experimental data are currently available to support this hypothesis.

The possible effects and implications of including LPMO enzymes together with other enzymes in the pre-treatment stages of biomass destructuration on the recovery of mineral nutrients have not been explored thus far, to the best of our knowledge. The focus has been mainly on using these enzymes in cellulose processing, mainly its saccharification and the production of nanocellulose and nanocellulose postmodification [104]. This is despite the already established fact that lignin boosts the LPMO activity, as mentioned above, so a pre-treatment with LPMO would make sense to apply from the first stages. It is probably related to the fact that the roles of these enzymes seem to not be completely elucidated. For example, just recently, they were shown to act not only on polysaccharides, but also on lignin and lignin–carbohydrate complexes [105]. This discovery brings a new perspective in understanding the usefulness of these enzymes along the entire process of biomass valorization, including in efficient pre-treatment and nutrient recovery. Taken together, it seems highly likely that the family of LPMO enzymes acts on more substrates than is currently known. There is also no information on how the minerals found in biomass influence the activity of these enzymes.

The liquid fraction from the hydrothermal pretreatment of wheat straw proved to be a good source of H_2_O_2_ and to boost the LPMO activity [106]. These discoveries support the idea that an efficient biorefinery would need several techniques that act on the substrate and potentiate each other.

Recently, Zhang et al. tested the bioleaching capacity of two fungi *Aspergillus niger* and *Fusarium oxysporum* and one bacteria, *Burkholderia fungorum* [107,108]. Significant bioleaching of some minerals from lignocellulosical biomass, such as sorghum straw, switchgrass, wheat straw, and corn stover, using the fungi *A. niger*, compared to water, *F. oxysporum*, and bacteria *B. fungorum*, was reported, with up to 80% mineral removal in the case of sorghum. The bioleaching efficiency depended on both the microbial strain and biomass type. The organic acids produced by the fungal strains are proposed to be responsible for the bioleaching. The final amounts remaining in the cultures upon microbial metabolization, which could be recovered and valorized, were not reported.

Besides the works mentioned above, the only other studies that discussed mineral recovery during biological and/or enzymatic pre-treatment are related to silica recovery, which will be discussed in Section 3. There is currently no other information, to the best of our knowledge, with respect to other mineral recovery using biological/biochemical approaches.

#### 2.2.2. Wet Treatments with Low Transition Temperature Mixtures (LTTMs)—Ionic Liquids (IL)

The utilization of ionic liquids (ILs) or room-temperature ionic liquids (RTILs) represents an alternative proposed approach to the classical pulp processes of biomass. This topic has gained tremendous interest in the last two decades, ever since the potential for innovative chemical technologies was realized, but the main focus has been on organic biomass fractionation and recovery, with much less concern on the mineral fraction of biomass. ILs have been known in one form or another since the turn of the 20th century. Still, they were relatively ignored for decades, until they started to be considered as a more environmentally friendly alternative to toxic, volatile, and flammable organic solvents. They were initially defined as salts with melting points below 100 °C, but the term is usually used nowadays to define solvents that consist only of ions. ILs are composed of large organic cations (imidazolium, pyrrolidinium, piperidinium, tetraalkylphosphonium, tetraalkylammonium, etc.) and inorganic or organic anions [109]. The main advantages over the classical organic solvents are the low volatility, thermal stability, and the unique advantage of having the possibility to design desired properties and task-specific solvents/cosolutes by selecting the proper cation/anion combination [110]. Certain ILs were reported to efficiently solubilize (hemi)cellulose [111,112], and several ILs were found to be suitable solvents for lignin [113,114], enabling its separation from hemicellulose and cellulose [115,116]. Due to these advantages, ILs were investigated and proposed as “green” solvents and co-solutes for a wide range of applications related to biomass valorization, recently reviewed in [117,118,119,120]:

✔ Pre-treatment, separation, derivatization, and/or fractionation of lignocellulose biomass [121,122,123,124];
✔ Wood processing technologies (reviewed in [125]);
✔ Biomass pre-treatment using ILs derived from lignin and hemicellulose [126];
✔ Pre-treatment followed by enzymatic saccharification of wheat straw [127,128] and other lignocellulosic biomaterials [129];
✔ Extraction of nanocellulose [130];
✔ Biodiesel production from waste cooking oil [131] and *Citrullus colocynthis* oil [132];
✔ In situ hydrolysis of empty fruit bunches combining pre-treatment and enzymatic hydrolysis [133];
✔ Extraction of natural compounds such as alkaloids, flavonoids, terpenoids, lipids, etc., reviewed in [134].

ILs can also act as catalysts for the conversion of biomass into value-added products, such as furfural, hydroxymethylfurfural (HMF), xylose, levulinic acid from (hemi)cellulose [135,136,137], phenolic compounds, dicarboxylic acids from lignin [117], etc.

Several groups also investigated the mechanism of lignocelullose dissolution in ILs. In the case of cellulose solubilization, the following results/conclusions were reported: the main forces responsible for cellulose dissolution in ILs are H-bonds [112]; the solvent should have a strong hydrogen bond acceptor (HBA) and a moderate hydrogen bond donor (HBD) [138]; the anions play a crucial role [138,139,140,141,142], but the chemical structure of cations has an influence also, with less agreement between scientists concerning their mechanisms, due to contradictory observations—reviewed and discussed in [141,142]; bulky heterocyclic structures and alkyl side chains might sterically inhibit the anions binding to cellulose; cation alkyl chains with electron-withdrawing groups were proposed to favor cellulose dissolution, based on molecular dynamics simulation [143]. Lignin solubilization in ILs presented some similar, but also distinct, characteristics, compared to cellulose: anions were also found to play an essential role, ILs with non-coordinating, large anions being poor solvents for lignin dissolution [113]; a minimum hydrogen bonding basicity was found to be necessary, although the hydrogen bond strength is not as crucial, as in the case of cellulose [144]; the presence of aromatic rings in the cation structure has a positive effect on lignin dissolution, due to π–π and n–π interactions with the phenyl aromatic rings of lignin [145] and references therein; the lignin–hemicellulose linkages are broken in ILs, and lignin is decomposed by the cleavage of ester, glycosidic, and β-O-4 ether bonds [116]; the solvation reaction rate depends on the cation–anion association [146]. Several groups have successfully used the β Kamlet–Taft and Hillebrand parameters to predict the pretreatment efficiency of ILs in lignocellulose dissolution; β ≥ 1 was correlated with increased efficiency and higher yields of fermentable sugars following pre-treatment [147,148].

From a separation point of view, the (hemi)cellulose and lignin should ideally have opposite solubility in a certain IL or, alternatively, two immiscible ILs with specific solubilizing effect towards (hemi)cellulose, and lignin could be used for good separation. Water and other protic solvents, such as ethanol and methanol, act as anti-solvents, even at less than 5 mass%, inducing lignin/cellulose precipitation and regeneration from IL, due to stronger H-bonding between water and IL than between –OH groups of lignin and IL, as shown in the case of lignin + 1-allyl-3-methylimidazolium chloride (AMImCl) [114] and cellulose + 1-ethyl-3-methylimidazolium diethyl phosphate ([EMIm] DEP]) [149]. On the contrary, specific quantities of aprotic solvents, such as dimethyl sulfoxide, dimethylformamide, and 1,3-dimethyl-2-imidazolidinone, improved the cellulose dissolution in [EMIm] [DEP] by 20–60% [149].

Despite the remarkable effects of ILs on biomass pretreatment, there are still several issues that should be addressed more in-depth, such as the high cost of some Ils and their recovery from anti-solvent mixtures, high temperatures needed (even > 140 °C), poor degradability and toxicity (which was shown to be significant for traditional Ils, such as the imidazolium-based ones that are some of the most effective in dissolving biomass), inhibitory effects on enzymatic activities, etc. [142]. Some of these drawbacks have already started to be addressed. Several groups designed and investigated cost-effective protic Ils from inexpensive chemicals that resulted in lignin removal and saccharification of cellulose [150,151]. Another promising approach is to design biomass-derived Ils, e.g., from lignin- and hemicellulose-derived aromatic aldehydes, which are both cost-effective and enzyme-friendly and could be integrated into a closed-loop process of biorefinery [126]. Xia et al. investigated the possibility of using aqueous Ils solutions for the pre-treatment of lignocelluloses and saccharification of (hemi)cellulose and found several effective agents [152]. The use of lower IL concentrations would be more cost-effective, but polar aprotic cosolvents should be used, as otherwise, it could result in low biomass solubility, as mentioned previously. Compressed carbon dioxide was reported to precipitate and separate the cellulose from IL-cosolvent mixtures [153]. Several groups reported the successful dissolution of cellulose at lower temperatures [154,155,156,157,158]. However, most of them required additional steps, the use of co-solvents, water washes, and/or were not tested on lignocellulosic substrates. The proper selection of IL and its concentration, based on understanding the interactions and mechanism [159,160], might lead to effective pre-treatment at mild temperatures and/or in aqueous ILs solutions [160]. Recently, effective pre-treatment of 10 wt% switchgrass at 50 °C using aqueous solutions of [TBA] [[OH] (tetrabutylammonium hydroxide), resulting in >90% glucose yield was reported [161].

Finally, one encouraging aspect is that ILs could be recovered and reused several times, as demonstrated for 8 and 5 cycles in the case of cholinium arginitate ([Ch] [Arg]) [124] and cholinium acetate (ChOAc) [162], respectively. These results are promising, but the investigations are still at an early-stage. Moreover, issues such as toxicity seem to be more challenging to overcome, although promising data were reported for certain amino acid-based ILs aqueous solutions at low concentrations of IL [163].

Although promising for lignocellulose destructuration and biomass valorization, there is limited information concerning mineral recovery from biomass when combining with ILs treatment, to the best of our knowledge, although the IL recycling process should benefit from the mineral recovery, and imidazolium-based ILs were shown to dissolve several minerals [164]. Zhang et al. removed minerals from switchgrass and a corn stover by treatment with 1-Butyl-3-methylimidazolium acetate ([C4mim] [Oac]), thus increasing the thermal stability of the biomass, but no further utilization for the recovered minerals was proposed. Recently, Shamshina et al. obtained chitin nano-whiskers with the simultaneous removal of mineral–protein matrix from crustacean biomass, using 1-butyl-3-methylimidazolium hydrogen sulfate [C4mim] [HSO4], again without any approach on the utilization of mineral fraction [165]. Considering that minerals seem to boost the degradation of ILs and be detrimental in the long run, as shown by Sarvaramini et al. [164], which would affect the ILs recycling, but which could also generate toxic and corrosive residues and complexes, it is not clear if and how the minerals extracted in ILs could, in fact, be valorized. A more optimal solution could be, in this case, the recovery of minerals by other methods prior to ILs treatment. This subject has not been previously addressed and needs in-depth studies.

#### 2.2.3. Wet Treatment—(Natural) Deep Eutectic Solvents, (Na)DESs

Lately, several groups have investigated and proposed a new, more environmentally friendly, and cost-effective approach for the extraction of lignin, minerals, and metabolites, as well as for cellulose processing [166]. Deep eutectic solvents (DESs), a relatively new topic in science, are considered as alternatives to ILs, or the “new generation of ILs analogs”, due to their similar physicochemical properties [167], although they are not entirely comprised of ionic species. They are generally synthesized by mixing two or more compounds (usually a salt (hydrogen bond acceptor—HBA)) with a hydrogen bond donor (HBD) in a certain proportion, with or without small percentages of water, until the mixture becomes liquid (recently reviewed in [168]). The melting point of DES is much lower than either of the individual components separately [169].

A subcategory of DESs is represented by natural deep eutectic solvents (NaDESs), which were synthesized mainly from primary metabolites, sugars, and amino acids [170,171]. They are more environmentally friendly and cost-effective than the traditionally highly expensive ILs [172]. The solubility and/or stability of some metabolites and complex biomolecules in NaDESs was much higher than in water [145,146,148], with polarity playing an important role. NaDESs were even proposed to play a role as alternative media to water in living organisms [173].

Similarly to ILs, the main interest in using (Na)DESs has been related to the fractionation and extraction of organic components of biomass, with less focus on the fate or recovery of minerals. Most of the research has been focused on the use of (Na)DES for the lignocelullosic organic biomass fractionation, with only recent concern about the influence of mineral fraction or its retrieval, mainly on silica. This part will be reviewed in the next chapter. Besides lignocellulosic substrates, (Na)DES was also investigated for the capacity to fractionate other types of biomasses, such as chitin-rich biomass (marine and insect biomass), algae, and fish by-products, including fish scales and bones, which are rich in hydroxyapatite.

In this section, we briefly overview the main applications of (Na)DES, and we discuss them together with the few studies that took into consideration the mineral issue, in the context of future perspectives of optimal biomass valorization.

During the last few years, more and more groups reported several (Na)DESs, mainly acidic ones, to be suitable solubilizing solvents for lignin [174,175] and for its extraction from lignocellulosic biomass, such as rice straw [176], corncob [177], corn stover [178], wood [179,180], wheat straw [181], and date palm [182], or good media for the electrochemical depolymerization of a lignin [183]. The high and low solubility of lignin and cellulose, respectively, in acidic (Na)DESs, such as formic acid: choline chloride (2:1), allowed the lignocelluloses components to be efficiently separated during biomass fractionation at 60 °C [184]. The acidic (Na)DESs have the drawback of being more toxic than the milder, weakly basic, or neutral (Na)DESs, which were shown to be less efficient for biomass pretreatment, producing moderate sugar yields [166]. Hou et al. proposed a two-step DES pretreatment, in which the two steps use different DESs, an approach found to improve the pretreatment efficiency [160] significantly. Their results also suggest that the presence of water could further improve the polysaccharide and/or lignin solubility in DES.

Some of the groups that investigated the lignocellulose dissolution in (Na)DES apparently obtained contradicting results: either lignin alone [176] or lignin plus hemicelluloses [180] were extracted by (Na)DES. One of the possible causes for the different results could be the parameters employed, such as temperature, HBD:HDA:water ratios, (Na)DES starting materials, substrate, etc. For example, lignin and hemicellulose were extracted in (Na)DES at much higher temperatures [180] than lignin [176] alone.

Other possible applications of (Na)DESs in biomass valorization reported lately were: the decrystallization of cellulose [185], development of polymer electrolytes based on cellulose acetate and DES [186], fabrication of nanofibrillated cellulose [187], production of cellulose films [188], dissolution of chitin [189], and production of chitin nanofibers [190], cellulose saccharification [191,192], extraction of bioactive natural products [171,193,194,195], and even wool deconstruction into functional, nano-dimensional α-keratin chains [196]. Some studies showed that the pretreatment of lignocellulose with (Na)DESs enhances the subsequent enzymatic saccharification, in terms of rate and extent [166].

To further reduce the cost of biomass valorization, (Na)DESs must be compatible with the various enzymatic activities employed during the process to avoid intermediate expensive regeneration steps. Only a few studies address this issue, such as the DESs-cellulase systems for lignocellulose hydrolysis [191,192,197,198]. In general, high concentrations of (Na)DESs were reported to inhibit the enzymatic activity; the higher the NA(DES) concentration, the lower the enzymatic activity. In some cases, cellulases were found to be tolerant [191,192], or even more stable and active in some (Na)DESs at moderate concentrations up to 30% [192,197], or even at 85% [198], compared to buffer. Lehmann et al. reengineered CelA2 cellulase by mutating one key residue, which resulted in increased enzymatic stability and activity in the NADES ChCl:Gly [199]. This approach should be investigated more in-depth. Special attention should be given to the pH of NA(DES)s, especially of those having an acid as HBD, which induces an acidic pH and deactivates the enzymes [198]; also low, viscosity solvents are preferred in the pretreatment process. Moreover, good enzymatic stability in a specific (Na)DES does not directly correlate with good hydrolysis performance of solid lignocellulosic substrates in that specific (Na)DES [198].

The biomass pretreatment and valorization in (Na)DESs has just started to be investigated, and much remains to be achieved and understood before the system can become economically feasible, but the concept seems to be promising. Future work should focus on testing more and elaborating on new (Na)DESs having high compatibility with the enzymes used in biomass valorization. Combining the high solubility of the lignocellulose components and high enzymatic activity in the specifically designed NaDESs would enhance the development of sustainable technologies for converting lignocellulose into valuable compounds. New solutions are needed for DESs recovery, solvent recovery and reuse, and zero-waste technology [197].

Although (Na)DESs are considered to be more environmentally friendly than the currently used solvents, due to the lower vapor pressure and benign constituents, not all are inherently “green.” The information reported lately by several groups indicates that the final DES product can have different properties than the individual constituents. Thus, some data indicated a higher toxicity on several bacteria and cytotoxicity on brine shrimp or Artemia salina leach of the DESs tested [200,201], but others found a similar or lower antibacterial/antifungal activity and toxicity on fish or *Hydra sinensis* [202,203], compared to the individual compounds or their aqueous mixtures. It was suggested that these apparently contradicting results could be due to the different species and different materials used in the respective studies [202]. More research is needed to clearly assign and understand the toxicological profiles of (Na)DESs.

As mentioned above, the mineral issue, when considering the biorefinery of lignocellulosic biomass, has been less approached, compared with the fate of the organic components. The mineral issue has started to gain some recognition relatively recently [204,205]. The recovery of minerals should be performed even before the (Na)DES treatment step, as it is believed that they interfere with (Na)DES recovery and extraction efficiency [204,205]. The few studies available mainly approached the silica recovery, as presented in the next chapter.

With respect to other types of biomass, (Na)DESs have been used for efficient hydroxyapatite extraction from fish scales, reviewed in [206], demineralization of chitin-rich biomass with promising results, for example up to 100% demineralization efficiency of crab shells in acidic DESs consisting of choline chloride and carboxylic acids (malonic, malic, or lactic acid) [207], more than 95% demineralization efficiency of insect powder with several, mostly acidic, DES [208].

Table 1 summarizes the main information from the previous studies related to the best performance of mineral recovery during the first stages of biorefinery.

## 3. Silicon Species Separation during the Initial Stages of Biorefinery

Very few studies were performed, until recently, for biosilica separation during the initial stages of biorefinery, despite the significant amount of biosilica associated with some plant biomass that could be used as feedstock in biorefinery processes. Most of the methods reported, as well as the most efficient methods, are based on chemical processes. Recovery of silica as a by-product from the beginning would have double benefits, further application of silica in different fields, including as plant biostimulants and the reduction or even elimination of problems induced by silica in subsequent biorefinery steps. Silica forms water-insoluble precipitates that can affect the industrial processes by blocking filtration systems and inducing instrumental defects. It can also complicates the further extraction and purification steps or recovery of solvents.

As mentioned in Section 2, silicon has been shown to be involved in covalent bonds with organic fractions in plant cell wall [56] and other references therein. The study proposes that the main interaction occurs between silicon and xyloglucan (XyG) and that it increases the recalcitrance to cellulase activities. This demonstrates that the understanding of the organization of plant biomass is still not complete and that there is still space for the biorefinery technologies to be improved. The biorefinery methods also targeting the full recovery of silica, purification of cellulose, and improvement of saccharification should be evaluated, in the context of the entire pre-processing of lignocellulose before the cellulose valorization. The mechanisms and all effects of different types of methods, either directly targeting the free mineral fractions or targeting the organic fractions, as well as those targeting the mineral-organic interactions, need to be understood and integrated. A relatively recent chapter summarized the technologies available for silica production from rice straw and husk [209]. The extraction and synthesis of silica nanoparticles, including from agro-waste, have also been reviewed recently by Yadav et al. [210]. Here, we shortly go through some of these technologies, emphasizing the process stage within a potential biorefinery technology and the involved environmental and economical issues. No single method is without disadvantages.

Table 2 summarizes several methods described, until now, for separating the biosilica from the biomass of the silicon-accumulating plants.

### 3.1. Dry Separation Processes

Calcination with or without chemical pre-treatment (alkaline or acidic, ultrasound) is the most commonly used method for mineral recovery, including silica [212,230], but it is a method that is applied at the end of the biorefinary process. In general, the dry separation consists of calcination that can be used at the end of a biorefinery process, after the separation of lignin, hemicellulose, and cellulose. A recent work reviewed the aspects related to the utilization of rice husk for energy and bio-silica production by combustion and analysis of the possibility of industrial scale-up of such a technology, in combination with suspension combustion, in terms of sustainability and self-reliance [26]. The problem with this approach is that it is capital intensive causes environment issues, and it does not usually consider the integration of significant steps of biorefinery for the recovery of other valuable compounds and the cascading approach included in the concept of biomass pyramid value [231]. Recovery of the bioactive components, such as polyphenols or proteins, should be considered before combustion. Calcination could be combined with microwave sintering, in case the reduction of amorphous silica is needed, plus the sol–gel method to induce nanoparticles formation, as reviewed by Setiawan et al. [232].

A dry silica recovery method that could be implemented in the first stage of biorefinery is based on mechanical forces, but only few studies are available, and it is not clear what the general impact on the total silica removal capacity and the downstream processes would be, see also review by Le et al. [24]. The only dry mechanical techniques previously investigated included hammering [228] and crushing [229], with the later being, in fact, part of sugarcane stalks processing for juice extraction. A third mechanical method, using ultrasonics, is usually performed in a liquid environment [226,227]. The methods were shown to be partially efficient, at least for the silica from the surface, but in some cases, acid hydrolysis or high temperatures were still needed. Hammering only removed up to 12.9% silica from oil palm empty fruit bunch, as reported by Law et al. [228]. No combination of hammering and ultrasonics have been reported until now.

### 3.2. Wet Separation Processes—Biological and Enzymatic Processes

Silicon solubilizing microorganisms could be important for lignocellulose biorefinery, the core of several bio-economy chains [233]. Yadav et al. have recently provided an in-depth review on various microorganisms that were shown to have the capacity of solubilizing silica from insoluble silicates or silicic acid from silica [210]. Many microorganisms, especially bacteria, were shown to have solubilizing capacity from inorganic precursors, and several mechanisms have been proposed: hydration of CO_2_ and formation of carbonic acid, complexation and chelation with microbial metabolites, or enzymatic mechanisms. This capacity of microorganisms is essential for the natural process called bio-weathering by which soluble silicon and other minerals are made available to plants. Despite this available information, only few studies that attempted to use microorganisms for silica removal from biomass are available, with most of them using fungal strains. Fungal silica bioleaching and biotransformation were demonstrated for *Fusarium oxysporum* [221], *Fusarium culmorum* [224], *Aspergillus parasiticus* [223], and *Trichoderma harzianum* [222]. The main issue of such biological pre-treatment with fungi is the decrease of the cellulose components [234], relative long processing time, and relatively moderate yield, with respect to more aggressive, chemical techniques. The method has the advantages of being more cost-effective and more environmentally friendly.

Prior desilication of lignocellulose during pre-treatment with microbial metabolites and enzymes acting as Si solubilizers (eventually in combination with lignocellulose weakening protein, such as cerato-platanins) should support a more straightforward bio-refining process. For this, it is, nevertheless, necessary to better understand the processes of silica bio-based solubilization, especially the enzymatic ones that are the least explored and that mostly lack experimental evidence. These involve silicases that hydrolase silica to silicic acid, specific reductases and oxidases, and other specialized enzymes. Combinations of these enzymes with those acting on the organic matrix could have promising outcomes. In the meantime, the microbial biomass resulting during metabolites production could represent active ingredients for microbial plant biostimulants integrated into the bio-economy value chains.

Besides microorganisms, biological pre-treatments based on vermicomposting that use worms have been investigated. For example, Californian red worms were grown for 5 months on rice husks, and the humus excreted by them was calcinated to obtain SiNPs [225]. A more in-depth study was performed by Torres et al., who used vermicomposting of different biomass types with red wiggler worms (*Eisenia foetida*) to obtain different types of SiNPs [235]. The growth period was shorter (2 month), and the efficiency (up to 90%) was higher than in the previous study. More details can be found in the review of Setiawan et al. [232]. Compared with the microbial alternative, this technology has the disadvantages of being even more time-consuming and of combining the biological methods with chemical and high temperature ones.

### 3.3. Wet Separation Processes—Chemical and Physico-Chemical Processes

This category of methods has been one of the widest reported by researchers for silica removal and recovery, as it involves the different possible approaches and combinations. Some groups reported recovery of silica by physico-chemical wet processes, e.g., a combination of alkaline and acid treatment [236,237] and sol–gel synthesis of nanosilica [216], recovery of silica from black liquor [238] or alkaline and autoclaving processes [239], organosolv pulping or solvothermal [240], with or without the additional Na_2_CO_3_ step, or even just Na_2_CO_3_ pre-treatment, in which case, the mixture of silica and lignin is obtained [218], as well as hydrothermal, superheated steam treatment (SHST) [241,242], high-pressure steam treatment (HPST), chelation [243], and references therein, sonochemical, laser ablation—see also review by Setiawan et al. [232], pyrolysis (cold) alkaline treatment combined with CO_2_ precipitation [217]. The source of CO_2_ could be the waste flue gas from an energy plant, biorefinery mill, or pulp mill chemical recovery circle, contributing to reducing greenhouse gas emissions [217]. Some of these methods have been previously reviewed in depth [24,232,244]. Other studies focused on the chemical recovery of silica from ash, i.e., after biomass burning/calcinations, using combinations of hydroxide and sulphuric acid, with the aim of reducing pollution with the silica generated from massive burning [245]. Although a step forward in the recovery of silica, these technologies make use of hazardous chemicals or need high temperatures or pressure, such as the organosolv pulping or consuming large quantities of water, and are still not environmentally friendly and/or cost-effective enough. Some of them are also difficult to implement in small-scale local biorefineries, due to the need for expensive equipment that also needs specialized training for proper operation. Moreover, there are not enough available studies, with respect to the effects on the following biorefinery steps, in the case of applying these methods as/during the pre-treatment of biomass waste.

Silica recovery was reported to be obtained only after lignocellulose extraction in ILs, by the standard dry method of calcination [214].

### 3.4. Wet Treatment—(Natural) Deep Eutectic Solvents, (Na)DESs

Although the main focus was not silica recovery, the co-extraction of silica in (Na)DES was observed in some studies. For example, silica was co-extracted together with lignin from paddy husks in a DES system consisting of ethylene glycol and citric acid. Further purification of silica from lignin was not reported [215]. In another study, a liquid–liquid biphasic lignin extraction with THF from the DES choline chloride–lactic acid used for rice straw fractionation facilitated the separation of ash rich in silica from lignin, resulting in lignin with 90% purity [246]. In a more recent study, silica was a co-extracted product in lignin isolated from wheat straw, with an alkaline DES composed of glycerol and K_2_CO_3_. Further fractionation by acidification down to pH 2 allowed for the separation of silica from lignin, resulting in a much purer lignin [247].

## 4. Phosphorus, Nitrogen, and Sulfur Recovery during the Biorefinery Process

As mentioned, the highest interest is to recover phosphorus (P) and nitrogen (N) from the biorefinery side streams. Sulfur (S) is recovered from the biorefinery, mainly due to its impact on biorefinery processes—(eco)toxicity and corrosivity [248]. Cationic nutrients are usually recovered together with their counter anions.

Currently, the P used in agriculture mainly comes from the six countries that have P-rich mines, with the top mining areas of phosphate rock being China, the United States, and Morocco (according to the U.S. Geological Survey (2012)). Phosphorous is mainly abundant in apatites, although it is present in many other minerals. Apatites have the chemical formula of Ca_5_(PO_4_)_3_(F, Cl, OH, Br) with the four forms denominated fluorapatite (the most common mineral), chlorapatite, hydroxyapatite, and bromapatite, respectively. Phosphate rock typically contains 13–17.5% P (or 30–40% P_2_O_5_, as often expressed for ore or fertilizer) and impurities such as heavy metals (cadmium, uranium, zinc, etc.) and humic acids (ref). Phosphorous is used in two main categories of applications: agricultural (95%) and non-agricultural. The agricultural applications are mainly in the fertilizer industry (90%), but also the production of animal feed supplements and phosphorus-based pesticides. The conventional fertilizers based on substances derived from nitrogen, phosphorus, and potassium are produced using various raw materials: natural gas and air for the production of ammonia; sulfur, coal, and phosphate rock for obtaining phosphorus components; and potash in the case of potassium.

It is estimated that population growth and continuing agricultural intensification will lead to the acceleration of P mines depletion. In the absence of P recycling, the agricultural productivity will not be able to sustain the world population [249,250]. Recovering and recycling P and N from wastewater and livestock waste has a double-positive impact: reducing over-fertilization in lakes and oceans and eutrophication of inland waters, as well as reducing the risks of P supply caused by war or embargo and mines depletion. The other benefits of recovery are: reducing the struvite fouling problems caused by anaerobic digestion, reducing CH_4_ emissions, and extending the useful landfill life by diverting organics from landfills [251]. Despite these benefits, only a small amount of P and N is currently recovered, due to its high cost/value ratio, compared to mined P. This ratio can be decreased by the simultaneous recovery of other valuable nutrients and minerals (N, K, Ca), high value metals (Ag, Au), organics, energy, and water [252].

There are currently two major methods of P agricultural and/or industrial reuse from wastewater and organic wastes: (1) direct land application of (processed by chemical precipitation, enhanced biological P removal (EBPR), incineration, etc.) sewage sludge/organic solids and (2) separation of P from wastewater, biomass solid waste, and incinerated sewage sludge ash (ISSA) by precipitation/crystallization, acid leaching, and/or thermochemical methods (recently reviewed in [251]). The second method is preferred, in order to have P in a plant to avoid the simultaneous unwanted land application of heavy metals that are concentrated in, for example, sewage sludge ash (SSA). There are two types of techniques for nutrient recovery from SSA, reviewed in [253]: thermochemical treatment and chemical extraction, including the electrodialytic separation (EDS) technique. The latter was recently shown to simultaneously separate heavy metals [254].

**Figure 2:**
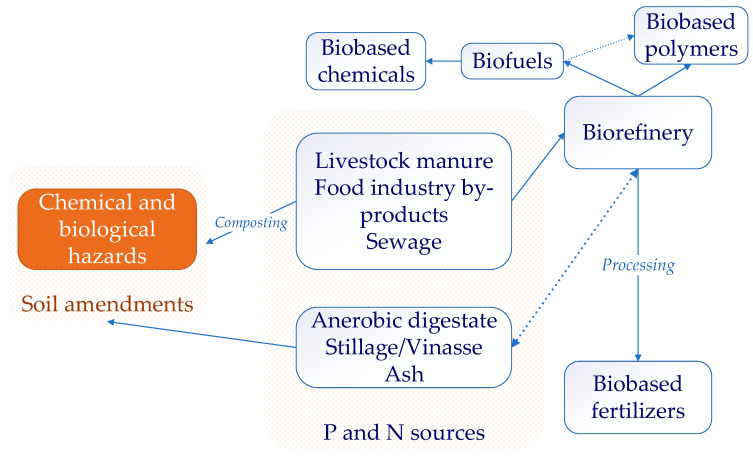
Advantages of the biorefinery approach that recover mineral nutrients as bio-based fertilizers and convert organic carbon into low-volume, high added-value products, including biodegradable bio-based polymers and biofuels that could be used as platform molecules for production of bio-based chemicals.

The significant sources for phosphorus (and nitrogen) recovery are livestock manure, food industry by-products (including animal slaughterhouse waste), and sewage [255]. By-products of biorefinery, e.g., anaerobic digestate [256] and (cellulosic) bioethanol vinasse/thin stillage [54], are other rich sources of phosphorus. For manure and food industry by-products, the biorefinery approach reduces contamination hazards—pathogens [257], antibiotics, and antibiotic-resistant genes [258]—and potentially toxic elements [259]. This biorefinery approach increases profitability by recovering the added-value products—nanocellulose and biogas/biomethane by anaerobic digestion [260]; organic acids from liquid (anaerobic) digestate further upgraded to bio-based biodegradable polymers, polylactic acid [261], polyhydroxyalkanoate [262], bio-oil, and hydrochar by hydrothermal conversion [263]. The processing of the biorefinery by-products by different technologies leads to bio-based fertilizers—Figure 2.

There are several investigated technologies that can be applied to the recovery of P, N, S, and other minerals from the by-products of the biorefinery process: pyrolysis and combustion, reverse osmosis (RO), phosphorous precipitation, (bio)electrochemical systems (BES), with or without forward osmosis (FO) or RO, etc.—Table 3.

In the following, we give a short overview of the state-of-the-art of some of the most investigated technologies. In the anaerobic digestion process nitrogen, sulfur and phosphate recovery are intrinsically related to the efficiency of the process [285]—and this will be presented in a separate sub-section.

### 4.1. Pyrolysis, Hydrothermal Carbonization, and Combustion

Pyrolysis represents the thermochemical decomposition of organic matter that results in gaseous (synthesis gas), liquid (bio-oil), and solid (biochar) products. The decomposition takes place in a near oxygen-free environment.

Combustion results in bottom and fly ash. The fly ash (from biomass combustion) represents the inorganic fraction that contains minerals, but also heavy metals. The bottom ash can be used as a fertilizer if it has not been contaminated, although it does not contain nitrogen. It has a high pH, due to high concentrations of alkali metals (Ca, K); therefore, it has to be distributed thinly. It should be applied in a “granulated” form, produced by mixing the ash with water and rolling it into small balls (developed in Scandinavia)—it is easier to handle and has a slow release of nutrients and reduced damage to ground vegetation (ref).

There are some biomass ash-related problems in renewable energy, such as the presence of alkaline metals and other minerals (K, Na, Cl, and P), which lower the melting point of the ashes during combustion and cause slagging and fouling problems, especially in superheater tubes, by forming complex eutectic salts.

Biochar, the porous carbonaceous solid produced by pyrolysis of the organic material in an oxygen-depleted atmosphere or by hydrothermal carbonization (HTC, also called hydrochar) [286], is an organic soil amendment that has received significant attention in the last two decades [287,288,289]. Biochar is considered a means to combat climate change and, at the same time, to achieve agricultural and environmental benefits [288,290].

Biochar, itself, has a poor nutrient value for plants [291]. However, biochar was demonstrated to adsorb ammonium and phosphate from side streams of the biorefinery/bioeconomy, especially from different wastewaters and gaseous and liquid emissions from composting [292,293]. The main mechanisms involved in ammonia and phosphate recovery from the aqueous stream were also analyzed and discussed [294,295]. The recovered phosphate and ammonium included in biochar are alternative slow-release fertilizers [292]. The characteristics of engineered biochar, with more functional groups on the surface, as an enhanced adsorbent for ammonium and phosphate from wastewater, were recently reviewed [296]. However, until now, there was not enough data to support the fertilizer efficiency of the biochar/hydrochar with recovered ammonia and/or phosphate from wastewater/compost streams [297,298]. The experiments on cultivated plants were performed with biochar that adsorbed phosphates or ammonia from the aqueous solutions, not from wastewater [299,300]. Biochar promotes fertilizer sustainable use [289]. However, further developments are still needed to utilize the potential of this approach for closing the loop of nutrients.

Ashes, officially called thermal oxidation materials derivates, are an efficient source of nutrients for cultivated plants in European soils [297,298]. The ashes resulting from biomass combustion include a variety of compounds, including various types of phosphorus compounds, with different bioavailability [298]. The standard methods used to predict the agronomic performance of soluble phosphate rock-based fertilizers are challenging for fertilizer phosphates from ashes [301].

The phosphorus bioavailability from biomass-derived ashes was increased by the wet extraction in acidic or base solutions. Some of the processes that produce phosphate fertilizers from biomass ash have been patented—Table 4.

When ISSA (incineration sewage sludge ash) is used as an ash source for phosphate recovery, the co-dissolution of (potentially toxic) metal(loid)s represents a major drawback [305]. Different solutions have been proposed. The evaluation of these solutions exceeds the scope of this work. In the last 5 years, several reviews of the technological, environmental, and/or economic assessment of P recovery technologies from sewage have been published [306,307,308,309].

Flue gases from biomass combustion must be cleaned according to the environmental requirements for nitric oxides [310], ammonia, and hydrogen sulfide [311]. Recently, a method to produce a slow-release fertilizer by combining fly ash from a biomass power plant with a solution resulting from flue gas denitrification and desulfurization was described [312].

### 4.2. Reverse Osmosis

Reverse osmosis is a membrane-based and pressure-driven filtration technique, in which an external applied pressure forces the solvent to move across a semipermeable membrane, from the high solute concentrated area to the less concentrated area, opposite to a normal (forward) osmosis (FO) process. The solutes are, therefore, concentrated on one side (rejected stream, retentate or brine) of the membrane, and the solvent (water) is accumulated free of solutes on the other side (permeate). Several groups investigated the RO-based nutrient and water recovery from different aquatic systems, such as wastewater [313,314] and biomass leachate [315,316]. The nutrient-rich retentate could be used as a liquid fertilizer. In the case of leachate, the high water/biomass ratio usually used (10–120) leads to high water volumes needed; therefore, water recirculation via processing technologies such as RO is important [316].

There are several issues with RO, when used as a stand-alone technique: it is energetically demanding, which induces high cost; all solutes, including the unwanted ones, such as heavy metals, are concentrated together—moreover, there is no control over the concentration of the different nutrients in the rejected stream. It is desirable that, at least certain nutrients, such as P and N, which can cause serious environmental problems, be recovered separately from other nutrients. Kumar et al. reported a more than 85% recovery of P from RO using polymeric ligand exchange (PLE), followed by precipitation as struvite, which can be used as a slow-release fertilizer [317]. Hybrid technologies involving RO and other techniques, which can overcome individual drawbacks, have emerged and are discussed below.

### 4.3. Phosphorous Precipitation

Phosphorus salt precipitation has several technologies that recover phosphorus as insoluble compounds—struvite/K-struvite, dittmarite, calcium phosphate, hydroxyapatite, and vivianite [297,318].

Struvite or magnesium ammonium phosphate (MgNH_4_PO_4_·6H_2_O) is a white crystalline compound which has low aqueous solubility under alkaline conditions. It can cause operational problems in waste treatment plants when unintentionally formed, but can also be used for nutrient recovery and as a slow-release fertilizer [22,319]. Struvite precipitation as a method of recovering N and P has been studied extensively [320,321,322]. It is usually induced and accelerated by additions of Mg^2+^ and high pH, although the latter proved to be controversial, based on struvite purity. Neutral pH induces a higher purity, but it significantly decreases the precipitation rate [323]; the authors suggested that alternative methods/compounds, such as calcium phosphate, should be taken in consideration for P recovery.

Several nutrient-rich streams, such as various sources of wastewater (leachate, urine, swine wastewater, industrial effluent, anaerobically digested sludge, etc.) and various methods (microbial fuel cells (MFC)), crystallization reactor with struvite accumulation device/airlift reactor, etc.), have been investigated, as reviewed in [324]. K-struvite (KMgPO_4_) can be used to simultaneously recover P and K [325].

Dittmarite (MgNH_4_PO_4_·H_2_O) is usually formed in side-streams with lower loads [326]. The technology to recover P as calcium phosphate utilises Ca(OH)_2_ as a precipitating agent and generates a material that is used as feedstock for fertilizer production or directly as slow-release fertilizer [318]. Hydroxyapatite is an efficient phosphorus fertilizer. The main drawback in hydroxyapatite precipitation technology is the microcrystals formation from the supersaturated solutions. Multiple feed ports and recirculation control this drawback [327].

There are two main limitations of nutrient recovery from wastewater via struvite precipitation: the low phosphorous concentration and the presence of heavy metals in wastewater. Alternative or complementary technologies involve membrane-based processes such as forward osmosis (FO), ED, and membrane distillation (MD). ED and MD were successfully applied for phosphorous [328,329] and ammonium [330] recovery, respectively. Nevertheless, these approaches come with their own challenges, such as the water permeability—solute selectivity tradeoff (FO), membrane wetting (MD), and fouling (ED and MD) [331,332]. One solution to improve nutrient recovery and to produce simultaneously high quality permeate water is to develop hybrid technologies by coupling complementary processes, such as FO and RO, FO and MD, FO and ED, ED and MD, ED and RO, or ED combined with struvite precipitation [332,333]. The integration of enhanced biological P removal (EBPR) with polyphosphate accumulating (micro)organisms (PAO) with phosphorus salt precipitation was recently reviewed [334].

### 4.4. (Bio)electrochemical Systems

The (bio)electrochemical systems (BES) are state-of-the-art technology in which organic wastes/compounds are used as electron donors (are oxidized at the anode) for microorganisms, a process that induces electricity production. Additionally, BES can be used to treat wastewater and to produce/recover value-added compounds [335].

The typical BES includes different configurations and designs: microbial fuel cells (MFCs) use micro-organisms as biocatalysts for converting the chemical energy of organics into electricity; microbial electrolysis cells (MECs)—hydrogen and methane production); microbial dialysis cells (MDCs)—chemicals and desalination; microbial electrosynthesis cells (MES).

MFCs are the most basic and the most investigated BES forms, being capable of generating energy and/or nutrient removal/recovery from different types of wastes, such as high- and low-strength wastewater, and probably animal waste and plant waste [335,336,337,338,339,340,341]. Wastewater remains, by far, the most suitable substrate for BES [342,343], but other substrates, such as landfill leachate, were also investigated [344].

Human urine is another waste that was successfully tested recently for BES-based nutrient (N, P, K, and Na) recovery by using MECs [345,346,347]. A recent work reported a new microbial electrochemical technology (MET) for the chemical-free and self-sustaining recovery of N, P, and K [345].

Nutrient removal/recovery has been facing more problems than energy production until recently: 27% of P from swine wastewater [348], 11.4% of N from urine [347], etc. Lately, higher removal/recovery rates, and even simultaneous N and P removal, were reported by using new/improved methods, such as the use of a recovery chamber between the cation and anion exchange membranes (technology termed a microbial nutrient recover cell (MNRC)) [349] or the use of both cation and anion exchange membranes in a closed circuit (method termed R^2^-BES) [350]. BES was also tested as a side or post-treatment of wastes used in other technologies, such as anaerobic digestion (AD), reducing the ammonia toxicity in AD [351]. Moreover, simultaneous electricity generation and ammonia recovery was obtained in recent studies [352,353]. Wu et al. managed to simultaneously recover N as the ammonia stripped in acid and produced hydrogen from rejected water (which has high ammonium concentration, but low concentration of biodegradable organics) in a bioelectrochemical reactor (BER). The system used influent wastewater (high in organics) to feed the anode and reject water for the cathode, where the protons were reduced to hydrogen gas and NH_4_^+^ was converted to NH_3_ [354].

Phosphorous is usually recovered through precipitation induced by the high pH resulting from the cathodic reduction reactions. A recent study reported a 96% P recovery from dewatering centrate using MECs, but the operation time had to be increased from 1 to 7 days [355].

BES has several advantages: it is less energy-intensive and more sustainable than traditional treatment, can use many substrates, such as waste/wastewater and natural sources, and has the possibility to use genetic and metabolic engineering to improve the performance [356]. The ion exchangers usually used are: cation/anion exchange membrane (C/A)EM, natural zeolite, ammonium selective polymer membrane, etc. [356].

Several challenges of nitrogen and phosphorous recovery/removal will have to be addressed: most of the current treatment processes focus rather on the removal than on the recovery of nitrogen from wastes [335]. New approaches for improving the N and P recovery rates are needed. Mathematical modeling technologies (engineering and statistics models) can be used to optimize the BES performance [357].

Osmotic bioelectrochemical systems (OsBES) are an emerging technology for the simultaneous recovery of nutrients, energy, and water (“NEW”) that has been lately proposed and investigated by several groups—as reviewed in [358]. The systems combine BES with FO. In FO, a concentrated draw solution and a diluted feed solution are separated by a semipermeable membrane. The permeation of water across the membrane is driven by the osmotic pressure difference. FO has been mainly investigated for its use as an alternative to RO in water desalination and treatment [359,360]. FO could increase nutrient recovery by concentrating the target compounds in wastewater [361] and assist with water recovery, while BES would improve FO by degrading the organic contaminants and providing a sustainable draw solute [362,363]. The number of studies related to nutrient recovery using OsBES systems is very limited [358,364,365], while others investigated nutrient removal, but not recovery [366,367]. The advantages of FO are its capacity of driving forces with high osmotic pressures that exceed the operating limits of RO, as well as its low fouling propensity and high rejection of contaminants from feed water [359]. Another reported approach is the integration of FO/OsBES with other existing resource recovering and complementary techniques, such as RO or anaerobic digestion (AD) [358,362]. The FO/OsBES membrane can improve the AD process for biogas production by pre-concentrating the wastewater and can harvest the nutrients from anaerobic effluent with simultaneous water recovery. Moreover, the ammonia recovery could reduce the inhibition of the AD process [352,353]. Several isssues, such as membrane fouling, salinity accumulation, and anaerobic treatment integration, need to be addressed more in depth [362]. Another important issue related to the OsBES system is the time coordination between FO (fast process) and BES (slow process). Some solutions were proposed by Qin et al., 2017 [358].

Several key challenges and issues will have to be addressed for implementing BES as a full-scale technology for water and nutrient recovery, as well as energy generation: toxicity of ammonia at high concentrations, volatile fatty acids, methane production (mainly in the case of animal waste), understanding microbiological processes, long-term operation and stability, system scaling up, operational cost, life cycle analysis, good control of pH, the effect of P precipitates on cathode performance, etc.

### 4.5. Nitrogen, Sulfur, and Phosphate Recovery during the Anaerobic Digestion

Anaerobic digestion (AD) is a well-established and eco-efficient technology that converts organic side streams into biogas and (anaerobic) digestates [5,368,369]. Anaerobic digestion integrates biorefineries in the circular bioeconomy [370,371]. AD valorizes the by-products of biochemical/microbiological biorefinery. Spent grains from bioethanol and alcoholic beverage production were reported to produce a high biogas yield [372,373,374]. Vinasse from bioethanol production is a suitable substrate for anaerobic digestion [375,376,377]. A total of 56.3% of pretreated and methoxylated biorefinery lignin was converted into methane [378]. The biological pretreatment of various organic side streams enhances biogas production [379]. The digestate could be further used in the biochemical biorefinery approach to producing biofuels and biochemicals [380]. The thermochemical conversion of digestate valorizes its high energy potential. Anaerobic digestion was integrated with microalgae cultivation [381,382]. Digestate was demonstrated to be suitable for the mixotrophic cultivation of microalgae [383,384,385]. Biogas upgrade to biomethane is feasible by cultivating microalgae in the liquid digestate-based medium for CO_2_ sequestration [386,387,388].

Some drawbacks limit the potential of anaerobic digestion. An illustrative example is the formation of hydrogen sulfide, a toxic gas, during the anaerobic digestion of sulfur-rich by-products [33], including those from the biochemical biorefinery, such as spent grains [389], stillage [390], or vinasse [391,392]. Hydrogen sulfide and its salts inhibit methanogenesis, due to high toxicity toward methanogenic consortia [393]. Ammonia formation is another hazard of anaerobic digestion, especially when nitrogen-rich substrates [394], such as pig slurry [395], chicken manure [396,397], or slaughterhouse wastes [398], are used. Ammonia also inhibits methanogenesis; however, the inhibitory concentration of ammonia, 2400 mg/L [394], is higher than in hydrogen sulfide, 50–400 mg/L [399].

**Figure 3:**
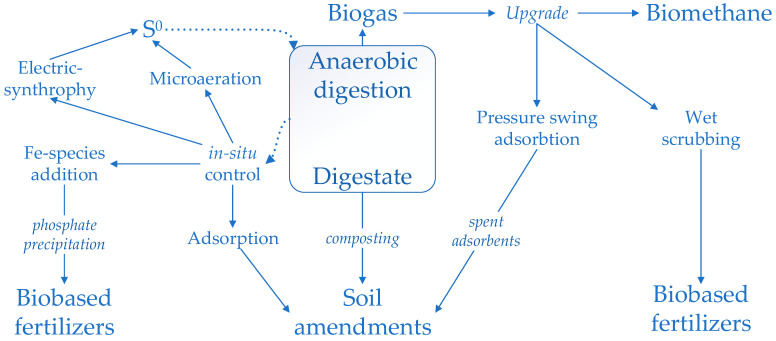
Potential of the technologies used to reduce hydrogen sulfide and ammonia in the biogas produced from anaerobic digestion to recover nutrients.

Two categories of technological processes are currently used to reduce hydrogen sulfide and ammonia in the produced biogas: (i) the purification of the gaseous phase [400], and (ii) the in-situ control of sulfide [393] and ammonia formation [401]. Both categories have a significant potential for eco-efficient and profitable mineral nutrient recovery—Figure 3. Nutrients are recovered either as soil improvers/soil amendments or bio-based fertilizers.

Ammonia stripping from anaerobic digestate and wet scrubbing in an aqueous solution of dihydrate gypsum (from a flue gas desulphurization plant) proved to be an economically feasible solution for upgrading biogas with simultaneous production of a bio-based fertilizer [402]. An operating cost of 5.8 euros per tone of treated digestate was determined, with a benefit of around 7 euros per tone of treated digestate [402]. Several commercial techniques for ammonia stripping through gas flow application were developed and recently reviewed, including the operating conditions [394]. An alternative technology that uses a thin film evaporator to transfer the ammonia from digestate to the biogas stream, coupled with fixation of the ammonia in the reactive adsorption unit, was tested at the pilot plant level [403].

Scrubbing hydrogen sulfide from biogas in aqueous solutions is hindered by high H_2_S toxicity and corrosivity [378]. The newly developed solutions to this technical problem, including the biological scrubbing systems, were recently systematically reviewed [404]. Patentable solutions were also proposed for this technical problem. One of these patent applications discloses a system that includes a vessel with a pressure release device and a scrubbing solution collector. The scrubbing solution is an aqueous solution of an aromatic nitrogen heterocyclic compound, hexahydro-1,3,5-tris(2-hydroxyethyl)-s-triazine. The resulting solutions containing sulfur species could be further converted to a sulfate fertilizer [405]. Another patent application is related to a process for producing ammonium thiosulfate (ATS) fertilizer simultaneously with biogas purification. The ammonia and hydrogen sulfide are stripped from anaerobic digester at a temperature higher than 90–95 °C, preferably up to 100 °C, and then reacted to generate ATS fertilizers [406]. Patent US10974190 B2 presents a cascading process, wherein hydrogen sulfide is initially fixed in a fixed bed of solid media, then transferred to a liquid media containing a sulfur dye catalyst, and the liquid media is oxidized by oxygen to produce thiosulfate from sulfide ions and to regenerate the sulfur dye catalyst. Thiosulfate solution is fed into an ion-exchange resin to produce potassium or ammonium thiosulfate, which is used as bio-based fertilizer [407].

The pressure swing adsorption technologies used for biogas purification rely on low-cost natural materials such as activated clays, zeolites, fly ash, biochar/activated biomass [408]. Almost all of these spent natural adsorbents could be further used as soil amendments.

Anaerobic digestate, itself, is often used as a soil amendment [409,410]. However, digestate has a low plant nutrient value and significant chemical (environmental) hazards, due to a high load of potentially toxic elements and high content of volatiles—ammonia, sulfides, and volatile fatty acids [411]. The biological hazards related to weed seeds, plant and entero-pathogens, and antibiotic-resistant genes are also significant [412]. Hybrid technologies that combine anaerobic digestion with aerobic composting were developed to improve the plant nutrient value and reduce the chemical and biological hazards [413].

One elegant solution to mitigate H_2_S emission risk from anaerobic digestion is to control the sulfide formation during anaerobic digestion—a recently reviewed subject [393]. These solutions protect methanogenic consortia from sulfide toxicity. In the meantime, some of these management approaches, which were developed to control the hazard of hydrogen sulfide formation, also influence the mineral nutrient recovery. The addition of Fe-species to anaerobic digestion modifies the interplay between the iron, phosphorus, and sulfur species during anaerobic digestion and promotes sulfide oxidation and phosphorus recovery in certain conditions [414,415,416]. Phosphorus recovery is enhanced by the addition of different forms of iron: rusty scrap iron [417], zero-valent iron nanoparticles (NZVI) [418], and steel slag [419]. Magnetite, stainless steel, and the addition of other conductive materials promote the direct interspecies electron transfer (DIET) between the exoelectrogenic bacteria (organic oxidizing bacteria, iron-reducing bacteria, sulfide oxidizing bacteria, and iron sulfate reducing bacteria) and electrotrophic methanogens [393,420]. A controlled DIET process determines the accumulation of zerovalent sulfur in the digestate [421], which increases its plant nutrient content. A similar effect, of increased formation and accumulation of zerovalent sulfur, is determined by the microaeration [422].

## 5. Critical Evaluation of the Methods used to Recover Mineral Nutrients during Biorefinery Processes

We described two large categories of technological solutions for mineral nutrients recovery from biomass during the biorefinery processes. The emerging ones relate to mineral nutrient recovery during the first stages of the biorefinery process. The mature technologies are those related to the recovery of agricultural nutrients from the biorefinery by-products.

**Figure 4:**
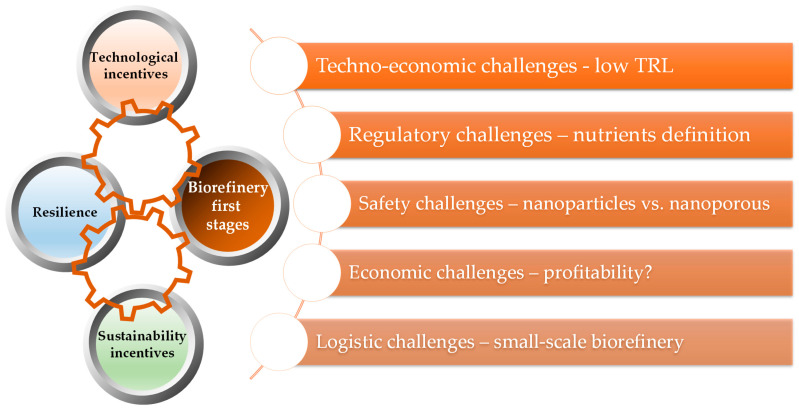
The incentives and challenges related to technologies that recover mineral nutrients in the first stages of the biorefinery processes.

The development of the methods that recover mineral nutrients in the first stages of the biorefinery processes is driven by technological incentives—the presence of minerals hampering the biorefinery process—Figure 4. As we mentioned, the silica embedded in the lignocellulose matrix enhances its resilience and generates silica scale and corrosion issues in industrial equipment [24]. Chitin/chitosan is recovered from crustacean shells after demineralization [423]. Biologically active fish collagen hydrolysate is produced from the fish bone after demineralization [277]. Phytic acid (inositol hexaphosphate) removal from rapeseed meal increases protein extraction yield [424].

The technologies that close the mineral nutrient loop from the beginning (initial biorefinery stages) are at the laboratory level. Phytic acid removal from rapeseed using phytase was demonstrated at the laboratory level [424] and analyzed as a potential business model [425]. Despite the significant content of minerals in rapeseed meal from biodiesel biorefinery [426], the recovery of mineral nutrients other than phosphorus has not been yet evaluated. The processes of silicon bioleaching from sorghum straws [107,108] or silica recovery from (Na)DES dissolved biomass fraction by the precipitation of cellulose with water, even on its patented version [427], are at low TRL. The further development of these technologies will enhance the sustainability of biomass biorefinery. A significant development is needed to support the use of the minerals separated in the first stages of the biorefinery process as ingredients for fertilizing products. Besides these techno-economic challenges related to low TRL, other challenges need to be addressed by the emerging technologies, targeted at the recovery of the nutrients in the first stage of biorefinery—Figure 4.

Regulatory challenges are among the most significant. The term “fertilizing products” relates to the regulatory framework for commercializing products made with recovered mineral nutrients. “Fertilizing products” include fertilizers, soil improvers, liming materials, growing media, and plant biostimulants [428,429]. Fertilizers (and the corresponding bio-based fertilizer, made with recovered minerals) must contain plant nutrients—and the plant nutrients, according to the current narrow definitions, are considered the only essential elements for plants [430].

Silicon, the principal mineral recovered during the lignocellulose material biorefinery, is not considered essential for cultivated plants [431,432]. Silicon does not fit the definition of an essential plant nutrient [430]. For example, there are no biochemical compounds with silicon—the silicon species in plant tissues are silicic acid and its polycondensation molecular species [433]. Despite the lack of biochemical compounds containing silicon, there is significant evidence related to the involvement of soluble silicon species in the plant secondary metabolism pathways related to plant pathogen responses [434]. Soluble silicon species are essential for plant responses to stress [435], interfering with plant endo-signals, including the phytohormones and oxygen-reactive species [436].

In natural ecosystems, plants sustain the silicon cycle during ecosystem retrogression [437]. Biogenic silicon (BSi), i.e., phytoliths/amorphous silica, replaces the lithogenic (LSi) and pedogenic (PSi) silicates [438]. The silicon species are moderately mobile species in soil solutions [439]. The agricultural practices decrease the bioavailable form of silicon, the biogenic silicon (BSi), by leaching BSi away from agricultural land, in a similar manner to nitrogen mobile species and soil BSi depletion, similar to phosphorus species with low mobility [440]. Several soil types (alfisol, entisol, histosol, inceptisol, oxisol, spodosols, and ultisol) were reported to have low available silicon [441]. Silicon-accumulating crops, such as sugar cane (*Saccharum officinarum*), rice (*Oryza sativa*), and wheat (*Triticum aestivum*), extract large amounts of silicon from soil [440], e.g., from 200 to 400 kg Si ha^−1^ y^−1^ in the case of rice [439]. To compensate for the BSi extracted from the soil, at least 80% of the rice straws must be returned to the soil [442]. Lignocellulosic biorefinery based on cereal straws has a risk of depleting the silicon in soils, especially in soil with low silicon, if active measures to return the biogenic silica to the soil are not implemented. Closing the loop for BSi within the lignocellulose biorefinery based on cereal straws will significantly increase the biorefinery sustainability.

Silicon is considered a beneficial element, and it is included in a class of agricultural inputs related to fertilizers and plant biostimulants [443], from the umbrella term “fertilizing products”, in European Union—Regulation 2019/1009 [444]. Plant biostimulants are defined by their agricultural functions—they enhance nutrient uptake and nutrient use efficiency, increase plant tolerance to abiotic stress, and improve crop quality traits [445]. Silicon effects on cultivated plants are similar to characteristics of plant biostimulants—and silicon is considered a plant biostimulant in EU countries [62]. The registration procedure before commercialization is more complicated for plant biostimulants than fertilizers, due to the field tests required to substantiate the agricultural function claims [429]. Fertilizers are easier to commercialize—usually based on a certificate of conformity stating the nutrient contents [446]. In other countries facing potential soil deficits due to soluble silicon extraction from their significant crops, products releasing soluble silicon species are considered fertilizers. Japan started, in early 1950, field trials with silicon fertilizers, followed by other Asian countries that rely on silicon-accumulating rice [447]. Brazil, a country that cultivates sugar cane (including for biorefinery purposes), ruled Si as an essential micronutrient since 2004 [441]. The recent proposal to enlarge the fertilizer class of agricultural inputs by including silicon (and other beneficial nutrients) among the mineral plant nutrients [61] will generate a framework that will promote the innovation and development of the process to recover BSi from biomass.

Cognitive barriers complicate the regulatory framework, especially in the case of recovered BSi. The advantages of recovery BSi as silica nanoparticles is considered significant for environmental sustainability [210]. Silica nanoparticles promote silicon uptake by plants because silicic acid, the most active silicon species in plants, is released at a higher rate from nanoparticles with a high surface volume ratio [448]. However, silica nanoparticles, even the amorphous nanoparticles recovered from natural sources, could have adverse effects on humans, leading, among others, to genotoxicity [449], adverse effects on human cardiovascular health [450], inflammation [451], and toxicity to the immune system [452]. Safety concerns should be considered in the development of technologies to recover BSi. The alternative nanoporous route could be considered because it combines a high surface volume ratio with a lower health hazard [453]. Logistic challenges are also significant. Small-scale biorefineries offer better opportunities to reuse mineral nutrients locally [454]. The pre-processing units (i.e., that recover minerals, such as BSi, and return them to the field), combined with centralized factories, were also demonstrated to be advantageous [454]. The possible combination of a small pre-processing unit and a small mobile biorefinery unit [455] presents additional advantages. Such future approaches are *win–win* solutions, improving biomass processability due to increased cellulose and hemicellulose accessibility and enhancing lignocellulosic biorefinery sustainability. The sustainability gain should sustain the improvement of the regulatory framework.

There are also challenges related to competition in the alternative use of recovered minerals as feed supplements or biomedical devices. The traditional method for chitin extraction from crustacean shells generates streams with high salinity, due to the combined utilization of HCl and NaOH. Greener extraction methods, using organic acid (HCOOH) and low doses of KOH, generate high-quality chitin and a potential plant fertilizer with calcium, potassium, and oligopeptides [456]. Recently, such technologies were reported to be upscaled to the pilot plant level—demineralization with citric acid and deproteinization with plant proteases [457]. For the resulting mineral by-product, calcium citrate alternatively used as a calcium supplement for humans and animals is envisaged [457]. Alternative biomedical utilization was considered for the hydroxyapatite recovered from fish bones [458]. However, the thermal conversion of fish bones into fertilizers and plant biostimulants is much easier to scale. The process was proposed to supply fertilizers in the least developed countries [459]. The circular value chains that close the mineral nutrients loop foster resilience and offer new opportunities for business redesign [425]. Despite the potential for improvement of biorefinery processes, the techno-economic analysis of the mineral recovery in the initial phases of the biorefinery processes has not been done yet, and the economic challenges are still unclear.

**Figure 5:**
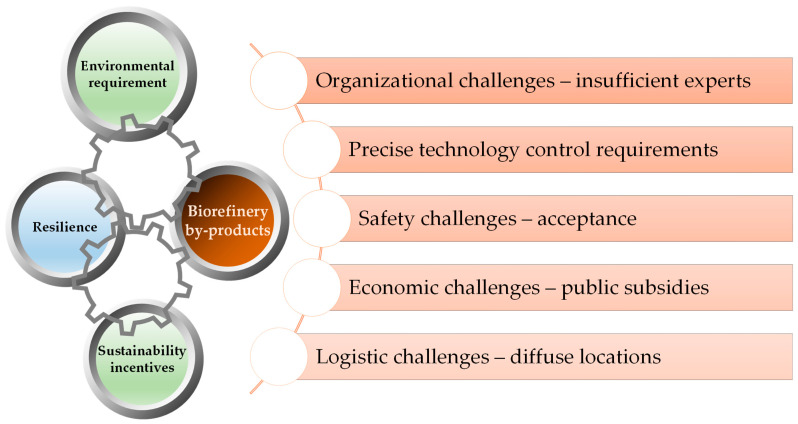
The incentives and challenges related to technologies that recover mineral nutrients from the by-products of the biorefinery processes.

In the developed countries, several methods that are used to recover nutrients from the biorefinery by-products have reached high TRL, and even commercial, levels. These methods are collectively called STRUBIAS, struvite, biochars, and ashes [298], and their development was driven by sustainability incentives and environmental concerns—Figure 5.

Phosphate rock is a non-renewable resource; by the end of the 21st century, almost 70% of the resources will be depleted [460]. Phosphorus pollution determines the water eutrophication affecting water quality from aquatic ecosystems [461] and having hidden negative effects on human health and economic activities related to aquatic ecosystems [462]. In the European Union, the interest in phosphorus recovery is also driven by a resilience dimension—phosphate rock is a critical material for Europe [463]. These environmental, sustainability, and resilience aspects related to phosphorus (and nitrogen) recovery from bioeconomy side streams are analyzed in detail in a handbook published in 2020, *Biorefinery of Inorganics* [464], that, despite its name, discusses only marginally the integration of this inorganic biorefinery in the biomass biorefinery.

The accumulating data show that biomass biorefinery has a significant P footprint. The production of corn bioethanol in China consumed 1% of the total mined phosphorus. In Brazil, phosphorus management, according to 5R (realign, reduce, recycle, recover, and redesign) method, could reduce by 63% the utilization of non-renewable phosphorus. The potential phosphorus recovery from corn bioethanol biorefinery from SUA is 3 times higher than from wastewater [465]. Anaerobic digestion converts organic carbon into CH_4_ and CO_2_ and concentrated minerals [466]. Phosphate recovery from the anaerobic digestate is a process that could compete at the limit, with the phosphate production from fertilizer rock considering phosphate market volatility [53] and future market development for other phosphorus applications, e.g., phosphorus 2D applications [467].

The existence of commercial solutions and the sustainability and resilience incentives push on the adaptation of the regulatory framework to allow for the commercialization of bio-based fertilizers, e.g., struvite. Variability of the natural feedstock determines a higher variability of the fertilizers based on recovered minerals. Regulation 2019/1009 includes provisions related to recovered fertilizers [444].

Organizational challenges influence the large-scale adoption of phosphate recovery technologies. The selection of the most suitable technology is difficult, due to insufficient experts. The phosphate precipitation process must be performed precisely in the precipitation area—struvite scale on pipes being a hazard associated with the precipitation technology scale-up [468]. Economic challenges are still significant, due to the phosphates market volatility, and phosphorus recovery subsidies are necessary to promote the technology adoption [469]. Although the existing regulatory framework insisted on contaminant control of the fertilizing product with recovered phosphorus, safety challenges translate into an end-user acceptance barrier [468]. Logistic challenges are also significant, due to the diffuse location of anaerobic digesters.

In an ideal situation, the two types of methods should be applied in a cascading approach. The recovery of the mineral nutrients in the first stage of biorefinery should focus on those minerals existing in large quantities that hamper biorefinery processes, e.g., silica. At the end of the process, mature technologies (such as phosphorus precipitation) should be applied on streams, wherein higher mineral nutrients are concentrated.

## 6. Agricultural Use of the Recovered Biofertilizers

One of the main issues related to fertilizer utilization based on recovered mineral nutrients from biorefinery side streams is the low acceptance by the farmers. In the case of fertilizers/plant biostimulants based on recovered biosilica (BSi), this low acceptance is due to the lack of knowledge related to the significant role of soluble silicon in stress agriculture [441]. Climate changes amplify the abiotic and biotic stress, and silicon increases plant tolerance to amplified stresses [470]. The lack of knowledge, regarding soluble silicon functions in the cultivated plants submitted to biotic and abiotic stress, is not only at the farmers’ level, but also at the expert level [441]. Some agronomists still consider silicon important only for silica-accumulating crops. The effects of silicon on non-accumulator plants, such as tomatoes [471], demonstrate that considering only silica-accumulating crops for products that release soluble silicon is a misperception.

Another knowledge gap is the lack of quality standards for silicon-based products and the standard method(s) for determining the bioavailable silicon in a given soil [441]. The BSi should be applied precisely according to specific pedo-climatic and crops in an ideal situation. Silicon accumulation has an energy cost, determining a trade-off between plant resistance against stress and plant yield [432]. Overdosing soluble silicon could determine yield reduction. The lack of analysis methods related to bioavailable silicon complicates implementing a precision agriculture system, which is needed to harness the BSi potential to increase agricultural systems resilience.

Low acceptance by the fertilizer industry and the farmers of the bio-based fertilizers is also related to contaminants, the low agronomical value of some recovered products, and unbalanced nutrient stoichiometry [21].

The mineral nutrients recovered from biorefinery, especially those based on recovered phosphorus, such as struvite, have chemical and microbiological hazards [472]. Concerns related to enhancing the resistome (antibiotic resistance genes) in the food chains was raised [473]. Struvite tends to concentrate copper—and copper accumulation in the soil promotes the spreading of antibiotic-resistant genes [474,475]. One solution to reduce these hazards associated with struvite is to use porous material, which reduces the mobility and bioavailability of the potential toxic elements, especially copper. It was demonstrated the biochar reduces copper bioavailability and antibiotic gene transfer [476]. Similar effects on the bioavailability of potentially toxic elements have also been demonstrated in natural siliceous nanomaterials, diatomaceous earth [477], and zeolite [478]. Such materials are characterized by a highly active surface, due to their nanoporous structure [63].

Another solution to the presence of contaminants is the application of recovered fertilizers that have high contamination hazards with nanoporous products recovered from biorefinery—biochar and nanoporous BSi. Biochar itself has a low nutrient content [297]. However, biochar (“Pyrolysis & gasification materials” and “hydrothermal carbonization material”) is a soil improver that reduces the bioavailability of some potentially toxic elements [479,480] that tend to accumulate in the “precipitated phosphate salts & derivates”. Combining biochar and fertilizer with recovered phosphorus could reduce the risks of contaminants.

There are many others benefits related to biochar utilization. Incorporating biochar in soils influences soil structure [481], soil texture [482], porosity [483], and wettability [484]. Published results show that biochar application reduced the leaching of nutrients from soil [485], reduced soil acidity [486], increased water retention [487,488,489], and reduced fertilizer requirements [490]. Adding biochar to soils increases crop yield and nutrient use efficiency, compared with control conditions [491,492]. Biochar application on soil increases plant tolerance to various abiotic stress [493]. Biochar application improves crop trait quality—especially in abiotic stress conditions, e.g., tomato under deficit irrigation [494] and rice under high-temperature stress [495]. Several biochar agricultural functions, i.e., enhanced nutrient uptake and nutrient use efficiency, increased tolerance to abiotic stress, and improved crop quality traits, are similar to the agricultural function considered characteristic for plant biostimulants [445]. Biochar regulates reactive oxygen species metabolism in a similar manner to plant biostimulants [496]. There is probably a significant contribution to the plant biostimulant-like effects of biochar on the cultivated plants results from the humic and fulvic acids included in the biochar [497,498,499]. The humic-like compounds are higher in hydrochar, compared to biochar [499]. Humic and fulvic acids are a representative class of plant biostimulants [500].

Nanoporous BSi is another solution to reduce potentially toxic elements (PTEs) bioavailability [501] and plant uptake [502]. Nanoporous silica materials immobilize PTEs, due to their high active surface area [63]. Soluble silicon reduces PTEs uptake by the plant root through several mechanisms [502]. An intermediary solution, until the development of BSi recovery from biorefinery stages to the commercial level (TRL9), is the use of diatomaceous earth. The use of phosphorus recovered from wastewater granulated with diatomaceous earth (DE) reduced the chemical and microbiological hazards associated with this biofertilizer [472]. DE is also a source of soluble silicon species—and soluble silicon species have a plant biostimulant effect on plants [62], including protection against abiotic stress [503], and have effects on soil microbiota [504], including on silicate solubilizing bacteria [505]. Stimulating silicate weathering also stimulates carbon fixation in the soil as a carbonate [506,507].

The agronomical value of the fertilizers made from recovered materials was analyzed in detail in a public document of the EU Commission [297]. Precipitated phosphate salts and the “K type” of ashes, rich in one or more macronutrients, especially in potassium and phosphate, proved their agronomical value [297]. The “K type” ashes are also an excellent raw material for purified ingredients, e.g., phosphate salts [255,428]. Purification/enrichment in one/two nutrients is also a solution to unbalanced nutrient stoichiometry.

**Figure 6:**
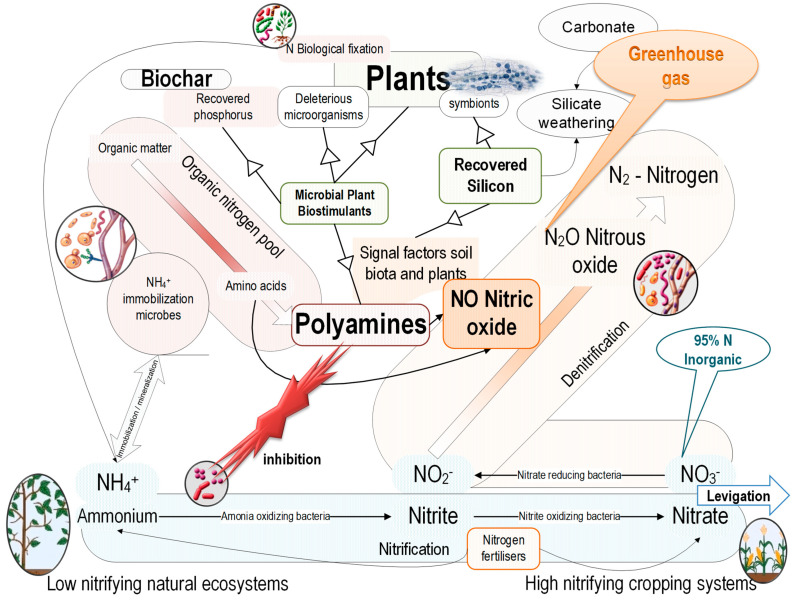
The contribution of the mineral nutrients recovered from biomass to carbon farming. Silicon recovered from biomass promote nutrient use efficiency and modulate soil microbiota, including the microorganisms acting for silicate weathering. Recovered microbial strain used for the production of the enzymes acting on biomass could be used to produce microbial plant biostimulants. Biochar promotes the sustainable use of recovered phosphorus.

The integrated use of recovered silica and biofertilizers with recovered phosphorus and with the nanoporous biochar and natural siliceous nanomaterials promote carbon accumulation in the soil by various mechanisms—Figure 6.

The target of such combined application is the establishment of a new equilibria in agricultural soil, intended to shift the agricultural systems toward the low nitrifying system. Maintaining the level of signal factors in the soil, such as nitric oxide and polyamines, at physiologically active thresholds and forming these signal factors from the organic nitrogen pool are significant elements of such low-nitrifying systems. The applied microbial biostimulants release these factors from the soil nitrogen pool. The microbial plant biostimulants are prepared from the strains used for the enzymatic cocktails needed in biochemical biorefineries. For example, *Trichoderma* strains are used for in-situ production of the enzymatic mixtures acting on lignocellulosic materials [508,509]. In the meantime, *Trichoderma* strains are beneficial plant microorganisms [510], and their ability to produce enzymes acting on the lignocellulosic matrix is significant for plant biostimulant effects [508]. *Trichoderma* efficient extracellular enzymes optimize the soil organic pool dynamics [511]. Another example is the *Bacillus* (sensu lato) group strains that produce cellulases tolerant to harsh conditions—ionic liquids [512] and high-temperatures [513]. The plant biostimulant effects of *Bacillus* strains were demonstrated to be related to their ability to produce polyamines [513,514]. Exogenous polyamines from soils stimulate the development of mycorrhizae symbiosis [515]. Arbuscular mychorrizae suppress the prokaryotes involved in the ammonia oxidation, the rate-limiting step of nitrification [516,517]. At the same time, arbuscular mychorrizae, stimulated by higher levels of polyamines in soil, promote carbon sequestration in soil [518,519].

Biochar application was also demonstrated to reduce nitrate and phosphate leaching. However, contradictory results of biochar application on the nitrogen cycle in different soil conditions were reported [520]. A meta-analysis from 2019, related to biochar influence on soil nitrogen species dynamics, concluded that biochar decreased by 13% NO_3_^−^ leaching and strongly reduced N_2_O-emissions in two types of soils, paddy and sandy soils [521]. Another meta-analysis, performed in 2021, found similar reductions in NO_3_^−^ leaching and N_2_O-emissions, with the simultaneous proliferation of nitrifying bacteria. The conclusion was that biochar reduced nitrogen emissions and leaching during the initial short-term applications, and long-term biochar applications would promote nitrate leaching and nitric oxide emissions [522]. Further research is necessary to understand the interactions between biochar application, soil (micro)biota, and soil microbiota signals. More investigations are still needed to understand biochar influence on microorganisms involved in N-cycling, in the short- and long-term, including on interactions with polyamines. The final goal is to re-establish low-nitrifying equilibria in the agricultural soil, which do not convert unbalanced nutrients in emissions and leakages.

The European Commission defined carbon farming as a business model that increases carbon sequestration in the agricultural soils, reduces greenhouse gases emission from the soil, and augments agricultural productivity [523]. Such a business model is fundamental for the sustainable soil health management considered by European Green Deal and its subsequent strategies—2030 Biodiversity Strategy [524], Farm to Fork Strategy [525], and Sustainable Carbon Cycles Strategy [526]. The support for this business model is the carbon farming scheme (CFS), in a multidimensional approach [527]. This multidimensional approach requires an absolute reduction in all types of greenhouse gases. The agricultural systems that promote soil carbon sequestration also stimulate nitrification and the subsequent release of nitrous oxide [528,529,530]. Nitrification inhibitors are a “double-edged sword” [15] that could promote ammonia emissions from soil [531].

The use of phosphorus recovered from wastewater granulated with diatomaceous earth (DE) not only reduces the chemical and microbiological hazards associated with this biofertilizer [472], but DE is also a source of soluble silicon species—and soluble silicon species have a plant biostimulant effect on plants [62], including protection against abiotic stress [503], and they have effects on soil microbiota [504], including on silicate solubilizing bacteria [505]. Stimulating the silicate weathering also stimulates a carbon fixation in the soil as a carbonate [506,507].

The potential utilization of recovered mineral nutrients from biorefinery for carbon farming represents another driving force for promoting sustainable loop closing in the bioeconomy value chains.

Another solution to the unbalanced nutrient stoichiometry is the integrated use of the recovered mineral in precision agricultural systems [532]. The integrated use of recovery minerals could promote integrated recovery from biorefinery processes, fostering biorefinery resilience and sustainability. Innovation ecosystems that reunite stakeholders (researchers, biorefinery experts, information and communication specialists, fertilizer industry engineers, professionals from distribution networks of fertilizers, farmers, authorities, and citizen representatives) present a working environment, wherein cross-fertilization between different types of knowledge generates solutions for optimal utilization of recovered mineral nutrients from biorefinery side streams. Such an innovative working environment should generate technological solutions accepted by the farmers, with a higher probability of adoption than the present one [533].

**Figure 7:**
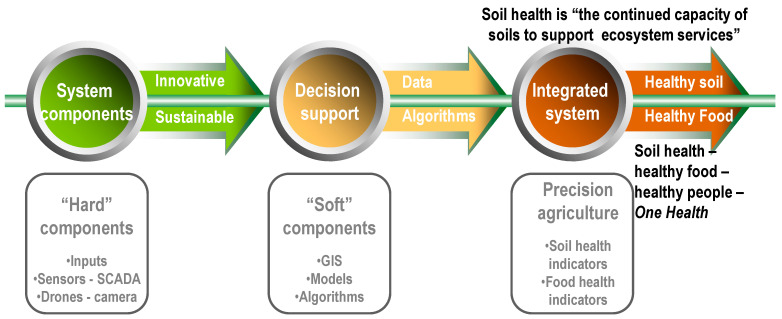
The digital dimension integrated into the precision agricultural systems supports the application of the recovered nutrients for enhanced soil health.

Such innovative ecosystems also integrate several essential dimensions for developing the agricultural utilization of recovered mineral nutrients. The business dimension substantiates a carbon farming scheme that provides additional benefits for farmers and sustainable incentives for fertilizers and biorefinery industries. The digital dimension is related to the precision farming approach [534]. It requires the development/optimization of the “hard” physical components of the systems (inputs, sensors for suction lysimeters, handheld laser-induced breakdown spectroscopy for in-situ soil analysis, low-power wide-area network of sensors and transmission stations, drones with hypercameras, light detection and ranging (LiDAR) sensors, etc.), integration with the soft components (models and algorithms, geographical information—GIS), and agrotechnical management practices in precision agricultural systems [535,536]. The precise agriculture systems resulting from integrating the components of the digital system support the application of recovered nutrients for enhanced soil health and global health—Figure 7.

The main goal of biorefineries is to produce value-added chemicals and bioenergy competitively, thus leading to a progressive replacement of oil refineries [537]. By integrating mineral nutrient recovery and the precise application of recovered nutrients, future biorefineries will generate resilient and sustainable circular value chains.

## 7. Conclusions

Integrated solutions to recover mineral nutrients should be applied in a cascading approach. In the initial stage of biorefinery processes, the target are the minerals hampering biomass fractionation and its component utilization—biosilica from lignocellulosic biomass, calcium carbonate, and calcium phosphate from the fish scale and fish bones. Phosphorus recovery from the biorefinery side streams is the most important, due to its sustainable and environmental dimension.

The recovery of silicon from biomass during the initial stage of the biorefinery is not yet significantly developed. However, its potential to improve biorefinery process and to generate additional benefits is significant.

Upgrading the regulatory framework to recognize silicon, a beneficial element for plants, among fertilizer components will boost the needed innovation to face the techno-economic challenges.

Phosphorus salt precipitation technologies (including the phosphorus extracted and precipitated from ashes resulting from the bioeconomy side-streams) are mature technologies with a cost competitive with the fertilizer produced from the phosphate rocks. The agronomical value of recovered phosphorus fertilizers is similar to that of the fertilizer produced from phosphate rocks. The contamination hazards limit the large-scale adoption of the recovered phosphorus. The application of the nanoporous structures produced during mineral nutrient recovery, e.g., nanoporous silica and biochar, together with fertilizers based on recovered phosphorus could control contamination hazards. Nanoporous silica and biochar reduce the bioavailability of potentially toxic elements.

Biosilica recovery is complementary to phosphorus recovery, and biochar generation forms highly recalcitrant biomass. Their combined application in the agricultural field benefits from microbial plant biostimulants, based on the strains used to produce enzymatic cocktails. The precise application of mineral nutrients recovered from biorefinery closes the sustainable and resilient circular value chain loop.

## Figures and Tables

**Table 1:** Investigated methods for mineral recovery during the first stages of biorefinery.

Mineral	(Possible) Biorefinery Step	Biomass	(Na)DES/Yield	Ref
General	Pre-treatment	Various types	(Dry) acid-based pre-treatment	Reviewed in [76]
General, mainly Cl, K, P, Mg	Pre-treatment	Sorghum, switchgrass, corn stover, wheat straw	Bioleaching with *Aspergillus niger*	[107]
Hydroxyapatite	Pre-treatment	Fish (*Aristichthys nobilis*) scales	ChCl:glycerol/>46%	[206]
Hydroxyapatite	Pre-treatment	Freshwater carp (*Carassius* sp.) scales	ChCl:1,4 butanediol/>40%	[206]
General, mostly CaCO_3_	Pre-treatment	Brown crab (*Cancer pagurus*) shell	ChCl:carboxylic acids (lactic, malic, malonic)/up to 100%	[207]
General, mostly CaCO_3_	Pre-treatment	Insect (*Hermetia illucens*)	ChCl, betaine:lactic, oxalic, butyric acid, urea, glycerol/up to 98%	[208]

Abbreviations: ChCl—choline chloride.

**Table 2:** Methods described until now for the separation of the biosilica from the biomass of the silicon accumulating plants.

Silica Form	(Possible) Biorefinery Step	Biomass	Method	Ref
Silica nanoparticles (NPs)	Final step	Rice husks	Acid pre-treatment/ Calcination	[211]
Mesoporous silica	Final step	Rice straw	Ultrasound-H_2_SO_4_ pretreatment + calcination	[212]
Silica NPs	Pre-treatment/ final step	Sugarbeet bagasse	Laser ablation	[213]
Porous silica NPs	Pre-treatment/ Final step	Rice husks	Lignocellulose extraction with ionic liquids + calcination	[214]
N/A	Pre-treatment	Paddy husks	Delignification with deep eutectic solvent (DES)	[215]
Spherical nanosilica	Pre-treatment	Rice husks	Nitric acid + NaOH + sol–gel synthesis	[216]
Insoluble colloid H_2_SiO_3_	Pre-treatment	Bamboo	NaOH cold extraction+ CO_2_ precipitation	[217]
N/A	Pre-treatment	Rice straw	Organosolv, Na_2_CO_3_	[218]
N/A	Pre-treatment	Oil palm empty fruit bunch (OPEFB)	Ammonia fiber expansion (AFEX)	[219]
Silica rosette-like microparticles	Final step	Pineapple peels	By-product of nanocellulose extraction	[220]
Nanocrystalline silica	Pre-treatment	Rice husks	Fungal-based bioleaching (*Fusarium oxysporum*)	[221]
Silica NPs	Pre-treatment	Rice husks	Fungal bioprocessing (*Trichoderma harzianum*)	[222]
Nanosilica	Pre-treatment	Rice husks	Fungal-based bioleaching and biotranformation (*Aspergillus parasiticus*)	[223]
Nanosilica	Pre-treatment	Corn cobs husks	Fungal-based biotransformation (*Fusarium culmorum*)	[224]
Nanosilica	Pre-treatment	Rice husks	Californian red worms	[225]
N/A	Pre-treatment	OPEFB, Sugarcane stalks	Mechanical (ultrasonic, hammering, crushing + size fractionation)	[226,227,228,229]

**Table 3:** Recovery of phosphorus, nitrogen, and sulfur and other nutrients from biorefinery side-streams.

Technological Solutions	Biorefinery Side-Streams	Resulted Fertilizing Product	Ref.
Pyrolysis	Distiller grains	Biochar with multinutrients, produced from distiller grains treated with wet-process phosphoric acid, neutralized with KOH, and pyrolyzed at 400 °C	[264]
	Distiller grains	Biochar composite, produced from distiller grains mixed with phosphogypsum that adsorb phosphate from water	[265]
	Anaerobic digestate from kitchen waste	Pyrochar (at 500 °C) with bioavailable phosphorus	[266]
	Anaerobic digestate of wastewater sludge and quinoa residues	Biochar that, at 25 t/ha, with liquid digestate (170 kg N/ha) increase by 25% growth of tomato plant	[267]
	Anaerobic digestate from commercial biogas producing plant	Pyrochar—soil amendment	[268]
Hydrothermal carbonisation	Sugarcane bagasse and vinasse	Hydrochar releasing nutrients and carbon, according to the sol and the applied dose	[269]
	Anaerobic digestate	Aqueous phosphate fertilizers, released from hydrochar by leaching with sulfuric acid	[270]
	Thin stillage	Hydrochar that collect the mineral nutrients from thin stillage	[271]
Combustion	Lignin from cellulosic ethanol	Ash rich in potassium and phosphorus	[272]
	Dried distiller grains	Ash rich in phosphorus that is available for Argentine canola (*Brassica napus* L.L. 5030) plants	[273]
	Distiller grains	Ash with high content of sulfur, nitrogen, phosphorus, and potassium	[274]
Reverse osmosis	Sugarcane vinasse	Organo-mineral fertilizer	[275]
	Anaerobic digestate from a commercial 2.5 MWe biogas plant	Liquid N/K-fertiliser, solid N/P-fertiliser, produced in a three-stage reverse osmosis unit, included in a pilot plant	[276]
	Anaerobic digestate from fish waste and cow dung	Concentrated liquid mineral fertilizer, solid fertilizer	[277]
Phosphorus precipitation	Concentrated cellulosic ethanol stillage	Struvite—MgNH_4_PO_4_·6H_2_O	[278]
	Vinasse, concentrate of the cathode area of the electrodialysis membrane	K-struvite—KMgPO4· 6H_2_O)	[279]
	Hydrolysate of microalgae, *Scenedesmus* sp.	Hydroxyapatite—Ca_10_(PO_4_)6(OH)_2_ Dittmarite—MgNH_4_PO_4_·H_2_O	[280]
	Liquid anerobic digestate	Struvite—MgNH_4_PO_4_·6H_2_O	[281]
	Thin stillage from corn ethanol	Ca-phytate, precipitated at pH 9.0 and 1.5:1 Ca-P ratio	[282]
Microbial fuel cell (MFC) and a Microbial electrolysis cell (MEC)	Anerobic digestate	Struvite—MgNH_4_PO_4_·6H_2_O	[283]
Microbial electrolysis cell (MEC)	Anerobic digestate	Struvite—MgNH_4_PO_4_·6H_2_O	[284]

**Table 4:** Examples of the patented solutions for producing phosphate fertilizers from biomass ash.

Treatment	Substrate	Operating Conditions	References
Dissolution in C1–4 monocarboxylic acid (formic, acetic, lactic, propionic, or butyric) solutions	Ash from fruits skin (hull), e.g., sunflower hulls	Solutions up to 35% organic acid, temperatures from 20 to 50 °C	US2022106237 A1 [302]
Dissolution of the phosphate-containing ash by adding nitric acid, separation of soluble phosphate fraction and soluble calcium fraction, precipitation of calcium nitrate, phosphate solution concentration	Sewage sludge ash (SSA) and/or meat-and-bone meal, plus phosphate rock	The rate between nitric acid and phosphate from the substrate is 1—to 1.8; phosphate-containing ash is dissolved at 50–60 °C for 70–80 min	EP3495323 B1 [303]
Mixing ash with a calcium or magnesium compound, followed by dissolution in phosphoric or sulfuric acid	Incinerated ash residue of chicken droppings	5–200 parts of calcium or magnesium compound mixed with 100 ash parts	US7452398 B2 [304]

## Data Availability

Not applicable.
